# Molecular genomic characterization of tick- and human-derived severe fever with thrombocytopenia syndrome virus isolates from South Korea

**DOI:** 10.1371/journal.pntd.0005893

**Published:** 2017-09-22

**Authors:** Seok-Min Yun, Su-Jin Park, Sun-Whan Park, WooYoung Choi, Hye Won Jeong, Young-Ki Choi, Won-Ja Lee

**Affiliations:** 1 Division of Arboviruses, National Research Institute of Health, Korea Centers for Disease Control and Prevention, Cheongju-si, Republic of Korea; 2 College of Medicine and Medical Research Institute, Chungbuk National University, Cheongju-si, Republic of Korea; National Institute of Infectious Disease, JAPAN

## Abstract

**Background:**

Severe fever with thrombocytopenia syndrome (SFTS) is an emerging tick-borne viral disease caused by the SFTS virus (SFTSV) from *Bunyaviridae* that is endemic in East Asia. However, the genetic and evolutionary characteristics shared between tick- and human-derived Korean SFTSV strains are still limited.

**Methodology/Principal findings:**

In this study we identify, for the first time, the genome sequence of a tick (*Haemaphysalis longicornis*)-derived Korean SFTSV strain (designated as KAGWT) and compare this virus with recent human SFTSV isolates to identify the genetic variations and relationships among SFTSV strains. The genome of the KAGWT strain is consistent with the described genome of other members of the genus *Phlebovirus* with 6,368 nucleotides (nt), 3,378 nt, and 1,746 nt in the Large (L), Medium (M) and Small (S) segments, respectively. Compared with other completely sequenced human-derived Korean SFTSV strains, the KAGWT strain had highest sequence identities at the nucleotide and deduced amino acid level in each segment with the KAGWH3 strain which was isolated from SFTS patient within the same region, although there is one unique amino acid substitution in the Gn protein (A66S). Phylogenetic analyses of complete genome sequences revealed that at least four different genotypes of SFTSV are co-circulating in South Korea, and that the tick- and human-derived Korean SFTSV strains (genotype B) are closely related to one another. Although we could not detect reassortant, which are commonly observed in segmented viruses, further large-scale surveillance and detailed genomic analysis studies are needed to better understand the molecular epidemiology, genetic diversity, and evolution of SFTSV.

**Conclusions/Significance:**

Full-length sequence analysis revealed a clear association between the genetic origins of tick- and human-derived SFTSV strains. While the most prevalent Korean SFTSV is genotype B, at least four different genotypes of SFTSV strains are co-circulating in South Korea. These findings provide information regarding the molecular epidemiology, genetic diversity, and evolution of SFTSV in East Asia.

## Author summary

Severe fever with thrombocytopenia syndrome (SFTS) is an emerging tick-borne viral disease caused by the SFTS virus (SFTSV). During entomological surveillance of SFTSV infection in Korean ticks collected from SFTS outbreak areas, we isolated a single SFTSV strain which we designated KAGWT. In addition, we isolated three SFTSVs from human patients with typical SFTS symptoms. In this study, we report the genomic sequences of each of these isolates and compare the genetic and evolutionary characteristics between tick- and human-derived Korean SFTSV isolates. Genetic and phylogenetic analyses of these sequences revealed that the tick-derived Korean SFTSV strain is clustered into genotype B, the most prevalent genotype in South Korea, and was closely related to other SFTSV in the same group. Furthermore, our results show that at least four different genotypes of SFTSV strains are co-circulating in South Korea.

## Introduction

Severe fever with thrombocytopenia syndrome (SFTS) is an emerging tick-borne viral disease characterized by fever, gastrointestinal symptoms, leukopenia, and thrombocytopenia. It was first reported in China in 2010 [1] and was subsequently identified in South Korea and Japan in 2013 [2–4]. The causative agent, SFTS virus (SFTSV), belongs to the genus *Phleboviru*s in the family *Bunyaviridae* [1]. Other novel tick-borne phleboviruses, including Heartland virus (HRTV), Malsoor virus (MV) and Hunter Island Group virus (HIGV), which are genetically related to but distinctly different from SFTSV, have been isolated from humans and ticks in the United States [5, 6], bats in India [7] and ticks in Australia [8], respectively.

Like other members of the genus *Phlebovirus*, SFTSV contains a tripartite RNA genome consisting of three single-stranded RNA segments of negative polarity, designated large (L), medium (M), and small (S). The L, M, and S segments encode the RNA-dependent RNA polymerase (RdRp), the viral envelope glycoproteins (Gn and Gc) and both a nucleoprotein (NP) and a nonstructural protein (NSs) in an ambisense orientation, respectively [1, 9].

Although human-to-human transmission of SFTSV through contact with blood and/or body secretions of patients has been reported [10–12], the virus is generally transmitted to humans by tick bites. Several studies have reported SFTSV isolation or detection from tick species including *Haemaphysalis longicornis*, *Rhipicephalus microplus*, *H*. *flava*, *H*. *concinna*, *Amblyomma testudinarium*, and *Ixodes nipponensis* [1, 13–18]. Further, this virus has also been isolated or detected from domestic animals (e.g., cattle, goats, dogs, chickens and cats), small mammals such as rodents and shrews [19–22], and reptiles [23].

Since the first reported fatal case in 2012 in Gangwon Province in South Korea [2], concern regarding SFTSV has grown as the numbers of SFTS patients has increased annually with 36 cases reported in 2013, 55 cases in 2014, 79 cases in 2015, and 165 cases in 2016 [24]. Moreover, the mean mortality rate of these cases was approximately 21.8%. During our previous survey of carrier ticks from affected areas, a single SFTSV strain (designated KAGWT) was initially isolated from *H*. *longicornis* nymphs collected from the Samcheok-si, Gangwon Province [15]. In addition, we also isolated SFTSV from two recovered- and one fatal-human cases that presented with typical SFTS symptoms. In this study, we analyzed the whole genome sequence of the first tick-derived Korean SFTSV and human SFTSV strains to compare the genetic and evolutionary characteristics between tick- and human-derived Korean SFTSV isolates. Genetic characterization revealed that the tick-derived SFTSV is closely associated with recent KAGWH3 Korean human isolates and that at least four different genotypes of SFTSV strain are co-circulating in South Korea. Taken together, our results suggest that more intensive and continuous surveillance of SFTSV is essential for better understanding of the molecular epidemiology, genetic diversity, and evolution of this virus in East Asia.

## Methods

### Ethics statement

Chungbuk National University Hospital received written consent for sample collection from each patient with SFTSV infection. All participants were adults and this study was approved by the institutional review board (IRB) of Chungbuk National University Hospital (IRB no. 2015-08-009-001).

### Virus propagation, cell lines, RNA extraction and cDNA synthesis

The KAGWT strain was isolated from *H*. *longicornis* ticks collected from the Samcheok-si, Gangwon Province in South Korea as described previously [15]. The CB1, CB2, and CB3 strains were isolated from the sera of SFTS patients who were hospitalized with typical SFTS symptoms at Chungbuk National University Hospital.

For virus propagation, the virus was passaged five times on confluent monolayers of Vero E6 cells (ATCC No. CRL-1586; American Type Culture Collection, Manassas, VA) in Dulbecco’s Modified Eagle Medium (DMEM; Gibco, Grand Island, NY) containing 8% fetal bovine serum (FBS; Gibco) with penicillin (100IU/mL) and streptomycin (100μg/mL; P/S, Gibco) placed in 37°C incubator supplemented with 5% CO_2_. Cell culture supernatant was collected after seven days and stored at -80°C as the working virus stock for whole genome sequencing.

Viral RNA was extracted from 140 μL of the viral stock using QIAamp Viral RNA Mini Kits (Qiagen, Hilden, Germany) according to the manufacturer’s instructions. Single strand cDNA was synthesized using viral RNA and primers specific for each segment using a cDNA synthesis kit (Cosmogenetech co, Ltd., Seoul, Korea) according to the manufacturer’s instructions.

### Whole genome sequencing by Sanger method

For the whole genome sequencing, one to six overlapping PCR fragments covering the SFTSV genome containing full-length L, M, and S segments were amplified by PCR from cDNA using SP-Taq DNA polymerase (Cosmogenetech, Seoul, Korea). PCR for each segment was performed at 95°C for 5 minutes, followed by 45 cycles of amplification consisting of 95°C for 30 seconds, annealing at 53 or 60°C for 30 seconds according to the primer sets for each segment, and 72°C for 2 minutes, with a final extension at 72°C for 5 minutes. The non-coding 5′ and 3′ ends of the viral genome were determined by rapid amplification of cDNA ends (RACE) method as described previously [25]. PCR products were then purified using a QIAquick Gel Extraction Kit (Qiagen) according to the manufacturer’s instructions and direct sequenced using ABI Prism BigDye Terminator Cycle Sequencing Kits (Applied Biosystems, Foster City, CA) and an ABI 3730x1 sequencer (Applied Biosystems) at Cosmogenetech Co, Ltd. The nucleotide sequences obtained from this study were assembled using the SeqMan program in the DNASTAR software (version 5.0.6; DNASTAR Inc., Madison, WI) to determine the complete genomic sequence.

### Genetic and phylogenetic analyses

Genetic and phylogenetic analyses were conducted by aligning published full-length sequences of SFTSV obtained from China, Japan and Korea isolates, which are available in GenBank, together with the closely related SFTSV sequences obtained from the basic local alignment search tool results. A total of 41 full-length sequences of the L, M, and S segments, including the strains isolated in this study (Table 1), were included in the analyses.

**Table 1:** Sequence information for SFTSV strains identified in this study.

SFTSV strain	Source of virus	Geographical origin	Year of isolation	GenBank accession No.			Genotype (L/ M/ S segments)^a^
				L segment	M segment	S segment	
KAGWT	*Haemaphysalis longicornis*	Gangwon Province, Korea	2013.6	KY273136^b^	KY273137	KY273138	B/ B/ B
CB1^c^	Human serum	Chungbuk Province, Korea	2014.8	KY789433	KY789436	KY789439	B/ B/ B
CB2	Human serum	Chungbuk Province, Korea	2015.7	KY789434	KY789437	KY789440	A/ A/ A
CB3	Human serum	Chungbuk Province, Korea	2016.10	KY789435	KY789438	KY789441	B/ B/ B

^a^ The SFTSV genotypes were classified according to the phylogenetic results of previous study [26].

^b^ GenBank accession number.

^c^ This strain was isolated from the serum of deceased human patient.

Multiple sequence alignments were performed using the Clustal W algorithm in DNASTAR version 5.0.6 or MEGA version 6.0 [27]. The aligned nucleotide and deduced amino acid sequences were analyzed using the MegAlign program of DNASTAR to compare the sequence homologies and amino acid substitutions.

Phylogenetic analyses were performed based on the full-length L, M, and S segments of SFTSVs. Phylogenetic trees were constructed with MEGA version 6.0 software using the Maximum Likelihood (ML) method based on the Kimura 2-parameter model. The reliability of the ML tree was evaluated by the bootstrap test with 1,000 replications.

### Accession numbers

The full-length L, M, and S segment sequences of tick- and human-derived strains determined in this study have been deposited in GenBank under the following accession numbers: KAGWT (KY273136 to KY273138), CB1 (KY789433, KY789436, and KY789439), CB2 (KY789434, KY789437, and KY789440), and CB3 (KY789435, KY789438, and KY789441) strains.

## Results

### Genome organization of tick- and human-derived Korean SFTSV strains

**Fig 1:**
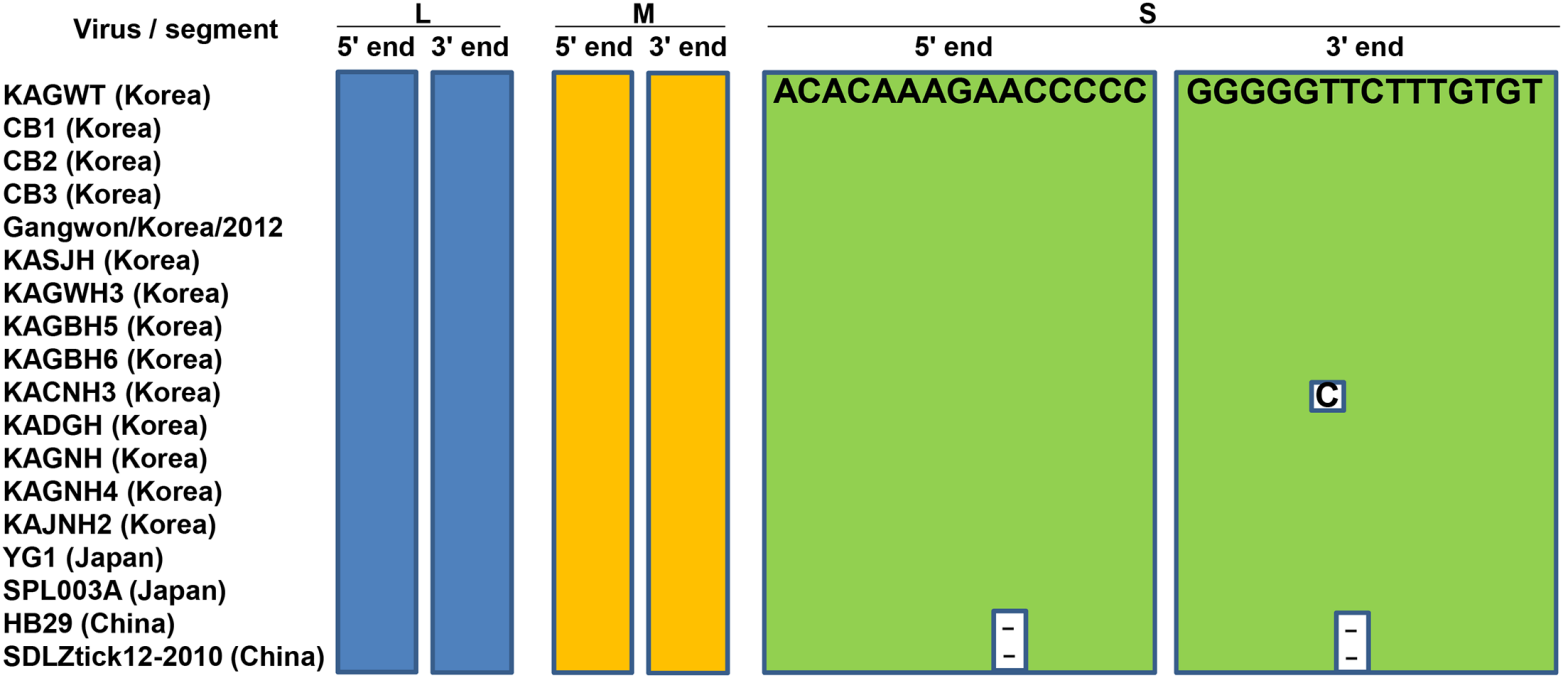
Nucleotide sequence alignment of complementary sequences within the 5′ and 3′-noncoding regions (NCRs) of L (blue boxes), M (yellow boxes), and S (green boxes) segments of 18 completely sequenced SFTSV strains, including tick- and human-derived Korean strains (KAGWT, CB1, CB2, and CB3). Compared with the sequence of the KAGWT strain, the different, and deleted sequences are indicated by open boxes, and hyphens, respectively. The closed boxes represent conserved complementary sequences within the 5′ and 3′ NCRs of the three segments.

The genomes of tick- and human-derived Korean SFTSV strains analyzed in this study were consistent with 6,368 nucleotide (nt), 3,378 nt and 1,746 nt present in the L, M and S segments respectively, consistent with what has been reported for other members of the genus *Phlebovirus* [9]. The L segment encodes a 6,255 nt long ORF [2,084 amino acids (aa)] for the RNA-dependent RNA polymerase gene, the M segment comprises a 3,222 nt precursor of the glycoprotein gene, coding for Gn (516 aa) and Gc (511 aa) proteins, while the S segment contains 882 nt and 738 nt long ORFs, which translate into a nonstructural protein (293 aa) and a nucleoprotein (245 aa), respectively. The non-coding regions (NCRs) of the L, M and S segments at the 5′ termini are 16 nt, 18 nt and 43 nt, respectively; and at the 3′ termini are 97 nt, 138 nt and 29 nt, respectively. As shown in Fig 1, complementary sequences within the 5′ and 3′ NCRs of the three segments are highly conserved between tick- and human-derived Korean SFTSV strains.

### Genetic analyses based on the complete L, M, S segment sequences of SFTSV strains

Alignment and pairwise comparisons of each segment between the tick-derived KAGWT strain and other fully sequenced human-derived Korean SFTSV strains showed nucleotide (and deduced amino acid) sequence homology ranging from 95.9 (99.3) to 99.9 (100), 94.0 (98.4) to 99.8 (99.8), and 94.9 (98.3) to 99.9 (100)% for L, M and S segments, respectively (Table 2). This high homology between the tick-derived KAGWT and other human-derived SFTSV strains reflects the close level of relatedness of these strains to each other.

**Table 2:** Comparison of the full-length nucleotide sequence (ORF region) and deduced amino acid sequence identities between the tick-derived KAGWT strain and other human-derived Korean SFTSV strains.

Genotype	strain	Segment							
		L		M		S (NP)		S (NSs)	
		Sequence identity (%)							
		Nucleotide	Amino acid	Nucleotide	Amino acid	Nucleotide	Amino acid	Nucleotide	Amino acid
Genotype A	CB2	96.3	99.5	94.0	98.5	95.8	99.6	95.0	99.3
Genotype B	CB1	96.6	99.5	96.2	99.1	95.7	99.6	95.9	98.6
	CB3	96.5	99.5	96.1	99.2	94.9	99.2	95.9	98.6
	KAGWH3	99.9	100.0	99.8	99.8	99.9	100.0	99.8	100.0
	KAGBH6	99.3	99.9	99.1	99.3	97.8	100.0	98.3	99.7
	KAGBH5	98.4	99.9	98.4	99.3	99.3	100.0	98.4	99.7
	KAGNH4	97.8	99.7	97.8	99.2	98.4	100.0	98.0	99.3
	KAGNH	97.5	99.8	97.3	99.3	97.0	100.0	97.1	99.7
	KAJNH2	96.7	99.7	96.2	99.1	95.8	100.0	96.1	99.3
	KADGH	96.5	99.4	95.8	98.8	96.2	100.0	96.5	99.7
	KACNH3	96.4	99.3	95.9	99.4	95.4	100.0	95.8	99.0
Genotype D	KASJH	95.9	99.7	93.9	98.5	95.7	99.2	95.1	98.3
Genotype F	Gangwon/Korea/2012	96.1	99.5	94.0	98.4	95.7	99.2	94.8	98.6

In particular, the KAGWT strain showed the highest sequence identities at the nucleotide and deduced amino acid levels with the KAGWH3 strain isolated in 2014 from the serum of a patient from Gangwon Province, South Korea who experienced typical SFTS symptoms [25]. These results indicate that tick- and human-derived Korean SFTSV strains are most closely related with one another. Moreover, the deduced amino acid sequence of KAGWT revealed two amino acid variations (A66S, I89V) in the Gn protein compared with the KAGWH3 strain. In particular, one unique amino acid substitution in the Gn protein, at position 66 (alanine to serine), was found only in the KAGWT strain compared with other human SFTSVs. According to previous reports, a change from phenylalanine to serine at position 330 in the M segment polyprotein (F330S) occurred in cell culture-adapted SFTSV and resulted in the large-focus phenotype [28]. However, all SFTSV strains analyzed in this study had a conserved amino acid sequence (F) at position 330 of the M segment.

Compared with the other fully sequenced SFTSV strains, KAGWT showed higher identity with the genotype B SFTSV strains than with the other genotypes at each segment as shown in S1 Table. The nucleotide sequence identities between the tick-derived KAGWT and other SFTSV strains from China, Japan, and South Korea belonging to the genotype B were 96.4 to 99.9% (L), 95.8 to 99.8% (M), 94.9 to 99.9% (NP) and 95.8 to 99.8% (NSs) similar, respectively. However, KAGWT showed relatively low nucleotide sequence identity ranging from 95.7 to 96.3% (L genes), 93.6 to 94.4% (M genes), 95.1 to 96.2% (NP genes) and 94.8 to 95.9% (NSs genes), respectively with other genotypes of SFTSV (Table 2 and S1 Table).

In comparison with the other fully sequenced SFTSV strains, CB2 was identified as the first genotype A SFTSV strain out of 14 full-length sequenced Korean SFTSV isolates. Genetic comparison results showed that the CB2 strain had nucleotide sequence identity ranging from 97.9% (L), 97.9 to 98.0% (M), 98% (NP) and 97.2% (NSs), respectively with the other genotype A SFTSV strains circulating in China. However, the CB2 strain showed relatively low nucleotide sequence identity ranging from 96.1 to 96.7% (L genes), 93.5 to 96.2% (M genes), 95.3 to 97.6% (NP genes) and 93.9 to 96.8% (NSs genes), respectively with other SFTSV strains (Table 2 and S1 Table).

### Phylogenetic analyses and genotype distribution

**Fig 2:**
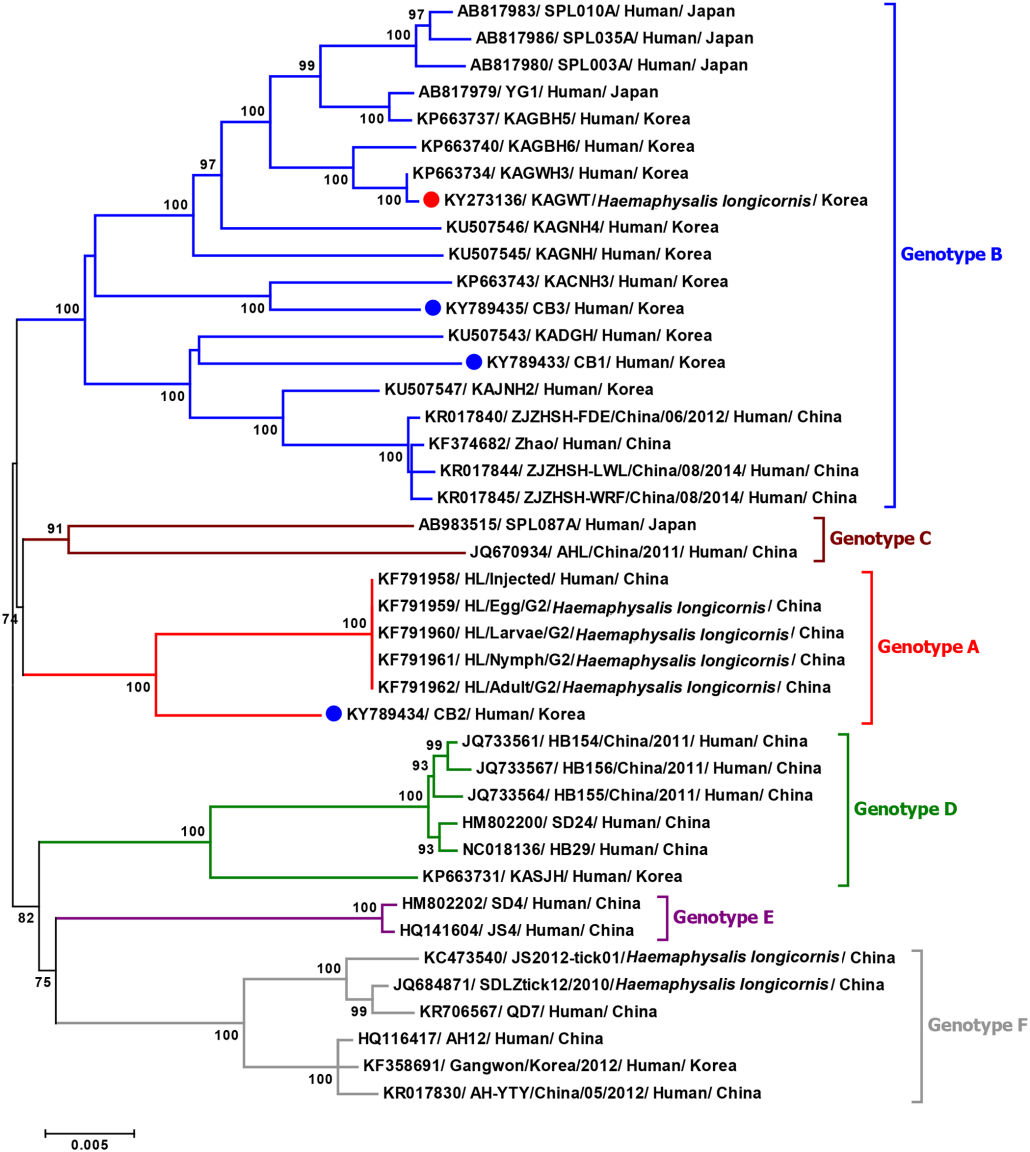
Phylogenetic analysis based on the complete nucleotide sequences of L segment of SFTSV strains using the Maximum Likelihood (ML) method based on the Kimura 2-parameter model. The numbers on the branches indicate bootstrap percentages based on 1,000 replications, and the scale bar indicates the nucleotide substitutions per site. The phylogenetic branches were supported with greater than 70% bootstrap values. The red, blue, brown, green, purple, and grey branches were designated as SFTSV strains belonging to the genotypes A, B, C, D, E, and F, respectively. The tick- and human-derived Korean SFTSV strains analyzed in this study are marked with red and blue closed circles, respectively.

**Fig 3:**
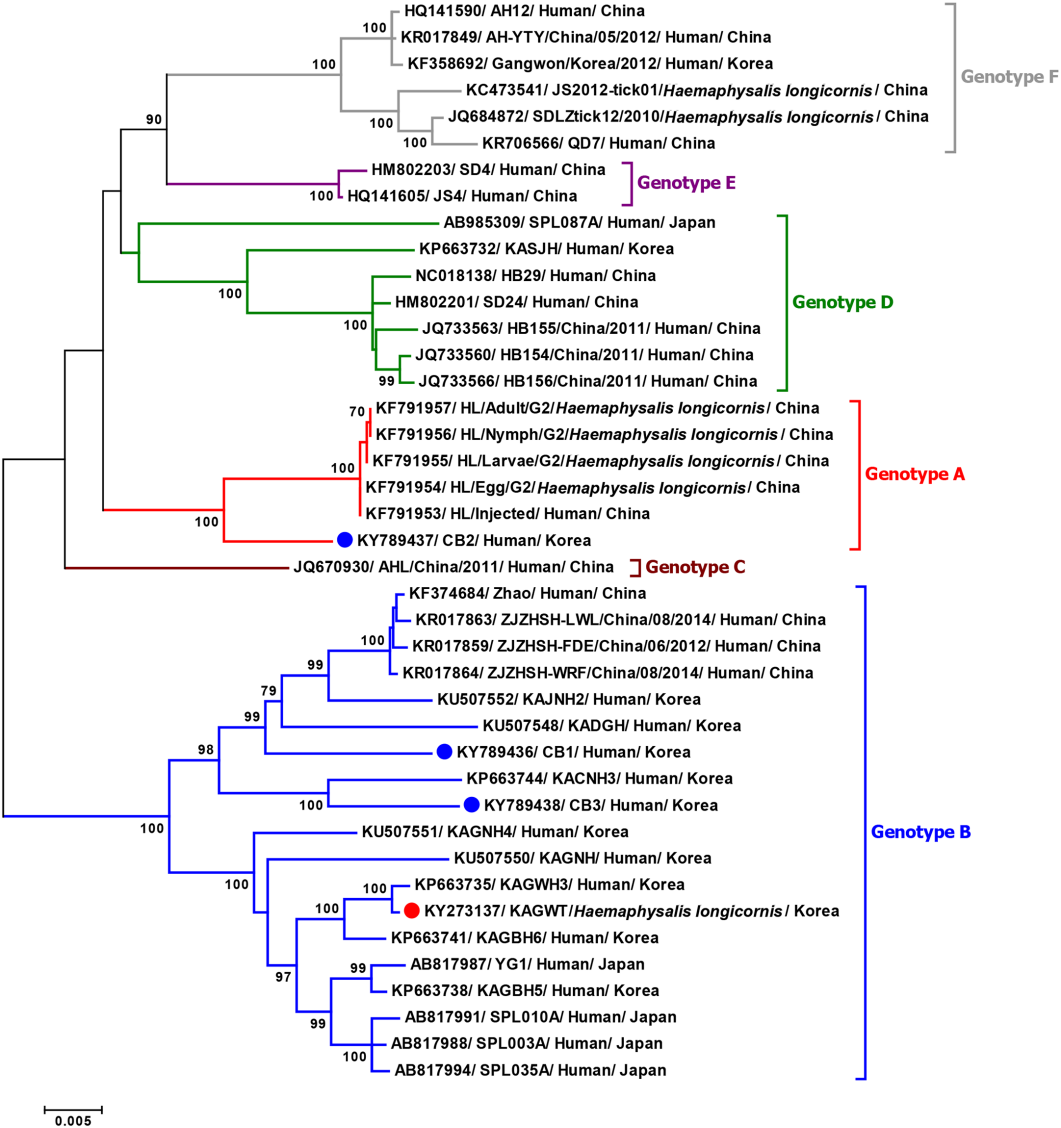
Phylogenetic analysis based on the complete nucleotide sequences of M segment of SFTSV strains using the Maximum Likelihood (ML) method based on the Kimura 2-parameter model. The numbers on the branches indicate bootstrap percentages based on 1,000 replications, and the scale bar indicates the nucleotide substitutions per site. The phylogenetic branches were supported with greater than 70% bootstrap values. The red, blue, brown, green, purple, and grey branches were designated as SFTSV strains belonging to the genotypes A, B, C, D, E, and F, respectively. The tick- and human-derived Korean SFTSV strains analyzed in this study are marked with red and blue closed circles, respectively.

**Fig 4:**
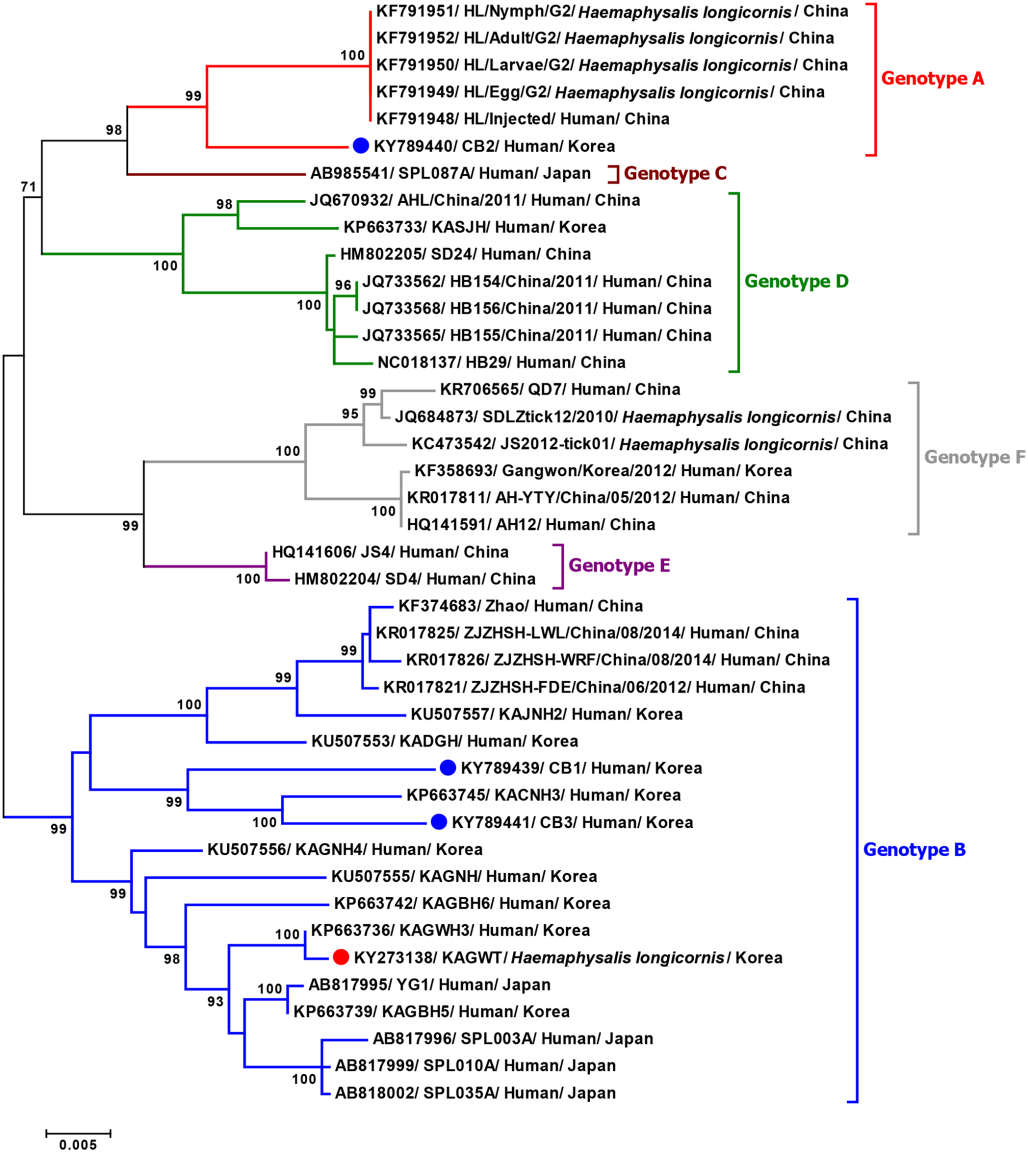
Phylogenetic analysis based on the complete nucleotide sequences of S segment of SFTSV strains using the Maximum Likelihood (ML) method based on the Kimura 2-parameter model. The numbers on the branches indicate bootstrap percentages based on 1,000 replications, and the scale bar indicates the nucleotide substitutions per site. The phylogenetic branches were supported with greater than 70% bootstrap values. The red, blue, brown, green, purple, and grey branches were designated as SFTSV strains belonging to the genotypes A, B, C, D, E, and F, respectively. The tick- and human-derived Korean SFTSV strains analyzed in this study are marked with red and blue closed circles, respectively.

To investigate the genetic evolutionary origins and relationship between the tick- and human-derived Korean SFTSV strains, phylogenetic analyses were conducted with full-length complete sequences of previous SFTSV isolates from China, Japan, and South Korea. Phylogenetic analyses of complete genome sequences (L, M, and S segments) indicated that the tick-derived Korean SFTSV strain (KAGWT) was clustered with the genotype B SFTSV strains circulating in humans in China, Japan, and South Korea (Figs 2–4), although KAGWT also exhibited a close relationship with the KAGWH3 human isolate [25].

Furthermore, the CB1 and CB3 human isolates also clustered with genotype B viruses although they are separated into a different sub-node from tick-driven Korean SFTSV strains (KAGWT) and are closely related with recent Korean SFTSV human isolates, KADGH and KACNH3. In addition, the CB2 human isolate was clustered together with recent China genotype A viruses.

Overall the phylogenetic trees revealed that Korean SFTSV strains can be classified with high branch support into four distinct genotypes (designated A, B, D, and F) out of the six genotypes described previously [26]. To investigate the prevalence of each genotype of SFTSV, we analyzed the SFTSV sequences available in the GenBank database. As shown in Table 3, most SFTSV strains from South Korea belong to genotype B (11 out of 14 isolates) and only one isolate each was reported from genotype A, D, and F. It is noteworthy that the CB2 is the first report case of a genotype A virus in South Korea. Isolates from China were diverse with all six genotypes being represented, although the most prevalent was genotype F followed by genotypes A and D (Table 3). In contrast, only three SFTSV strain genotypes (B, C, and D) observed in Japan and the most prevalent genotype was B.

**Table 3:** Genotype distribution of SFTSV strains isolated from China, Japan, and South Korea.

Geographical origin	Genotype	Number of SFTSV strains belonging to each genotype / Total number of SFTSV strains^a^		
		L segment	M segment	S segment
China (n = 139)	A	30/139	30/139	31/139
	B	17/139	17/139	17/139
	C	1/139	1/139	0/139
	D	26/139	21/139	26/139
	E	2/139	2/139	2/139
	F	63/139	68/139	63/139
Japan (n = 43)	B	42/43	42/43	42/43
	C	1/43	0/43	1/43
	D	0/43	1/43	0/43
South Korea (n = 14)	A	1/14	1/14	1/14
	B	11/14	11/14	11/14
	D	1/14	1/14	1/14
	F	1/14	1/14	1/14

^a^ In total, 196 SFTSV strains containing full-length sequences of L, M, and S segments were included in the analysis.

## Discussion

SFTS is an emerging tick-borne infectious disease caused by a novel *Phlebovirus*, SFTSV that is highly endemic in China, Japan, and South Korea [1, 2, 4].

In this study, we determined the whole genome sequences of the first tick and patient-derived Korean SFTSV strains and compared them with other available whole genomic SFTSV sequences. To the best of our knowledge, this is the first report of the whole genomic sequence of an SFTSV strain isolated from ticks and of a genotype A SFTSV strain collected from South Korea. Comparison of the whole genome sequence of tick- and human-derived strains revealed that all SFTSVs consist of three segments with 6,368 nt in the L segment, 3,378 nt in the M segment, and 1,746 nt in the S segment. Thus, the genome organization of Korean strains are consistent with the known genome organization of SFTSV belonging to the *Phlebovirus* genus [9]. Complementary sequences within the 5′ and 3′ NCRs of the three segments analyzed in this study are highly conserved, as in other SFTSV strains (Fig 1). According to previous studies of Bunyaviruses, these complementary sequences form panhandle-like structures and may have important roles in transcription initiation, viral RNA replication, viral RNA encapsidation, and viral genome packaging [29, 30]. All Korean SFTSV strains have 53 unique amino acid variations in the L, Gn, Gc, N, and NSs proteins compared with other SFTSV strains. Among them, one unique amino acid substitution in the Gn protein at position 66 (alanine to serine) was found only in the tick-driven KAGWT strain. Since little information is known about SFTSV, additional research regarding the association of amino acid variations in each gene product with the pathogenesis of SFTSV infection and detailed molecular studies utilizing reverse genetics will be required to further explain the functional role of these unique substitutions and in particular, the Gn protein substitution (66S).

Genetic and phylogenetic analysis revealed that the tick-driven KAGWT strain isolated from *H*. *longicornis* nymphs in 2013 [15] has high sequence identity in each segment and is closely related with the KAGWH3 strain isolated in 2014 from the serum of an SFTS patient who experienced high fever, vomiting, diarrhea, and fatigue. This is not surprising given that both viruses were from the Samcheok-si, Gangwon Province [25], and further suggests that tick- and human-derived Korean SFTSV strains are closely related to each other.

Although several studies about SFTSV classification have been reported [26, 31–34], the uniform classification of SFTSVs has not yet to be established. Therefore, to use unified nomenclature of SFTSV genotypes, more mutual understanding and discussion are needed. In this study, we adapted the nomenclature as previously described by Fu *et al*. [26] which described results of most large numbers (205 SFTSV strains) and complete sequences. That paper showed SFTSV strains can be divided into six distinct genotypes. Through full-length sequence analysis of human-driven SFTSV isolates we detected the first genotype A strain (CB2) from a patient exhibiting severe SFTSV-like symptoms in the Chungbuk Province of South Korea in 2015. The Korean strains belong to four of these (genotypes A, B, D, and F) of which genotype B strains appear to be predominant. It should be noted that in China all six genotypes of SFTSV have been reported, while only one isolate was reported for each genotype A, D and F in Korea. Thus, further detailed surveillance of tick- and human-derived SFTSVs are needed to understand the actual prevalence and possible transmission of each genotype of SFTSV strain into South Korea. Due to the segmented nature of the *Phlebovirus*, genetic reassortment is known to be an important process resulting in the sequence diversity necessary for viral evolution [35]. Previous studies have shown reassortment occurs in Phleboviruses including Hantavirus [36, 37], Rift Valley fever virus (RVFV) [38], and SFTSV [26, 31–34]. Although SPL087A and AHL/China/2011 SFTSV strains isolated in Japan and China, respectively, were identified as reassortants which contained different genotype segments in the same SFTSV strain [26, 31–34], no reassortant was identified in the Korean SFTSV strains tested in this study. Therefore, continuous analysis consisting of in-depth surveillance and genome sequencing will be needed in South Korea to obtain more detailed information of the molecular epidemiology, genetic diversity, and evolution of SFTSV.

In conclusion, we report here the first whole genomic sequence of the tick-derived SFTSV KAGWT strain isolated from the ticks in the Gangwon province of South Korea and the comparison of the genetic characteristics of this virus with recent human SFTSV isolates. Genetic and phylogenetic analyses with full-length genome sequences revealed that tick- and human-derived Korean SFTSV strains are closely related to one another and that at least four different genotypes of SFTSV are co-circulating in South Korea. These results provide insight into the genetic origins of human of SFTSV strains as well as shed light on the molecular epidemiology, genetic diversity, and evolution of SFTSV. Furthermore, these associations will have important implications for the design of diagnostic procedures and vaccines against SFTSV.

## Supporting information

S1 Table Nucleotide sequence (ORF region) and deduced amino acid sequence identities of (A) L, (B) M, and (C and D) S segments of SFTSV strains belonging to the different genotypes. Nucleotide identities (%) are shown above the diagonal and the deduced amino acid identities (%) are shown below the diagonal. 1, CB2 strain; 2, HL/Injected strain; 3, HL/Egg/G2 strain; 4, HL/Larvae/G2 strain; 5, HL/Nymph/G2 strain; 6, HL/Adult/G2 strain; 7, KAGWT strain; 8, KAGWH3 strain; 9, KAGBH5 strain; 10, KAGBH6 strain; 11, KACNH3 strain; 12, KADGH strain; 13, CB1 strain; 14, CB3 strain; 15, KAGNH strain; 16, KAGNH4 strain; 17, KAJNH2 strain; 18, YG1 strain; 19, SPL003A strain; 20, SPL010A strain; 21, SPL035A strain; 22, Zhao strain; 23, ZJZHSH-FDE/China/06/2012 strain; 24, ZJZHSH-LWL/China/08/2014 strain; 25, ZJZHSH-WRF/China/08/2014 strain; 26, SPL087A strain; 27, AHL/China/2011 strain; 28, KASJH strain; 29, HB154/China/2011 strain; 30, HB155/China/2011 strain; 31, HB156/China/2011 strain; 32, SD24 strain; 33, HB29 strain; 34, JS4 strain; 35, SD4 strain; 36, Gangwon/Korea/2012 strain; 37, SDLZtick12/2010 strain; 38; JS2012-tick01 strain; 39, AH12 strain; 40, QD7 strain; 41, AH-YTY/China/05/2012 strain.
(DOCX)
