# Supplementary material for: Molecular genomic characterization of tick- and human-derived severe fever with thrombocytopenia syndrome virus isolates from South Korea
Source: PLoS Negl Trop Dis. 2017 Sep 22;11(9):e0005893. doi: 10.1371/journal.pntd.0005893 (PMC5627960; doi:10.1371/journal.pntd.0005893)
Supplement: S1 Table — Nucleotide identities (%) are shown above the diagonal and the deduced amino acid identities (%) are shown below the diagonal. 1, CB2 strain; 2, HL/Injected strain; 3, HL/Egg/G2 strain; 4, HL/Larvae/G2 strain; 5, HL/Nymph/G2 strain; 6, HL/Adult/G2 strain; 7, KAGWT strain; 8, KAGWH3 strain; 9, KAGBH5 strain; 10, KAGBH6 strain; 11, KACNH3 strain; 12, KADGH strain; 13, CB1 strain; 14, CB3 strain; 15, KAGNH strain; 16, KAGNH4 strain; 17, KAJNH2 strain; 18, YG1 strain; 19, SPL003A strain; 20, SPL010A strain; 21, SPL035A strain; 22, Zhao strain; 23, ZJZHSH-FDE/China/06/2012 strain; 24, ZJZHSH-LWL/China/08/2014 strain; 25, ZJZHSH-WRF/China/08/2014 strain; 26, SPL087A strain; 27, AHL/China/2011 strain; 28, KASJH strain; 29, HB154/China/2011 strain; 30, HB155/China/2011 strain; 31, HB156/China/2011 strain; 32, SD24 strain; 33, HB29 strain; 34, JS4 strain; 35, SD4 strain; 36, Gangwon/Korea/2012 strain; 37, SDLZtick12/2010 strain; 38; JS2012-tick01 strain; 39, AH12 strain; 40, QD7 strain; 41, AH-YTY/China/05/2012 strain. (DOCX) [file pntd.0005893.s001.docx]

| **Sequence homology (%)** | | | | | | | | | | | | | | | | | | | | | | | | | | | | | | | | | | | | | | | | | | |
| --- | --- | --- | --- | --- | --- | --- | --- | --- | --- | --- | --- | --- | --- | --- | --- | --- | --- | --- | --- | --- | --- | --- | --- | --- | --- | --- | --- | --- | --- | --- | --- | --- | --- | --- | --- | --- | --- | --- | --- | --- | --- | --- |
| **Genotype** | | **A** | | | | | | **B** | | | | | | | | | | | | | | | | | | | **C** | | **D** | | | | | | **E** | | **F** | | | | | |
|  |  | **1** | **2** | **3** | **4** | **5** | **6** | **7** | **8** | **9** | **10** | **11** | **12** | **13** | **14** | **15** | **16** | **17** | **18** | **19** | **20** | **21** | **22** | **23** | **24** | **25** | **26** | **27** | **28** | **29** | **30** | **31** | **32** | **33** | **34** | **35** | **36** | **37** | **38** | **39** | **40** | **41** |
| **A** | **1** | **—** | **97.9** | **97.9** | **97.9** | **97.9** | **97.9** | **96.3** | **96.3** | **96.4** | **96.3** | **96.2** | **96.3** | **96.1** | **96.5** | **96.1** | **96.4** | **96.5** | **96.4** | **96.2** | **96.2** | **96.1** | **96.6** | **96.6** | **96.5** | **96.5** | **96.5** | **96.2** | **96.5** | **96.2** | **96.2** | **96.2** | **96.2** | **96.2** | **96.4** | **96.4** | **96.7** | **96.4** | **96.4** | **96.7** | **96.2** | **96.6** |
|  | **2** | **99.7** | **—** | **100.0** | **100.0** | **100.0** | **100.0** | **96.1** | **96.1** | **96.2** | **96.1** | **96.0** | **96.1** | **95.9** | **96.3** | **96.0** | **96.2** | **96.3** | **96.2** | **96.1** | **96.0** | **96.0** | **96.3** | **96.3** | **96.2** | **96.2** | **96.3** | **96.0** | **96.2** | **96.0** | **96.0** | **96.0** | **96.0** | **96.0** | **96.1** | **96.1** | **96.5** | **96.3** | **96.3** | **96.5** | **96.1** | **96.4** |
|  | **3** | **99.7** | **100.0** | **—** | **100.0** | **100.0** | **100.0** | **96.1** | **96.1** | **96.2** | **96.1** | **96.0** | **96.1** | **95.9** | **96.3** | **96.0** | **96.2** | **96.3** | **96.2** | **96.1** | **96.0** | **96.0** | **96.3** | **96.3** | **96.2** | **96.2** | **96.3** | **96.0** | **96.2** | **96.0** | **96.0** | **96.0** | **96.0** | **96.0** | **96.1** | **96.1** | **96.5** | **96.3** | **96.3** | **96.5** | **96.1** | **96.4** |
|  | **4** | **99.7** | **100.0** | **100.0** | **—** | **100.0** | **100.0** | **96.1** | **96.1** | **96.2** | **96.1** | **96.0** | **96.1** | **95.9** | **96.3** | **96.0** | **96.2** | **96.3** | **96.2** | **96.1** | **96.0** | **96.0** | **96.3** | **96.3** | **96.2** | **96.2** | **96.3** | **96.0** | **96.2** | **96.0** | **96.0** | **96.0** | **96.0** | **96.0** | **96.1** | **96.1** | **96.5** | **96.3** | **96.3** | **96.5** | **96.1** | **96.4** |
|  | **5** | **99.7** | **100.0** | **100.0** | **100.0** | **—** | **100.0** | **96.1** | **96.1** | **96.2** | **96.1** | **96.0** | **96.1** | **95.9** | **96.3** | **96.0** | **96.2** | **96.3** | **96.2** | **96.1** | **96.0** | **96.0** | **96.3** | **96.3** | **96.2** | **96.2** | **96.3** | **96.0** | **96.2** | **96.0** | **96.0** | **96.0** | **96.0** | **96.0** | **96.1** | **96.1** | **96.5** | **96.3** | **96.3** | **96.5** | **96.1** | **96.4** |
|  | **6** | **99.7** | **100.0** | **100.0** | **100.0** | **100.0** | **—** | **96.1** | **96.1** | **98.4** | **96.1** | **96.0** | **96.1** | **95.9** | **96.3** | **96.0** | **96.2** | **96.3** | **96.2** | **96.1** | **96.0** | **96.0** | **96.3** | **96.3** | **96.2** | **96.2** | **96.3** | **96.0** | **96.2** | **96.0** | **96.0** | **96.0** | **96.0** | **96.0** | **96.1** | **96.1** | **96.5** | **96.3** | **96.3** | **96.5** | **96.1** | **96.4** |
| **B** | **7** | **99.5** | **99.5** | **99.5** | **99.5** | **99.5** | **99.5** | **—** | **99.9** | **98.4** | **99.3** | **96.4** | **96.5** | **96.6** | **96.5** | **97.5** | **97.8** | **96.7** | **98.4** | **98.1** | **98.2** | **98.0** | **96.6** | **96.7** | **96.5** | **96.6** | **95.9** | **95.7** | **95.9** | **95.7** | **95.7** | **95.7** | **95.7** | **95.7** | **96.1** | **96.0** | **96.1** | **96.0** | **96.0** | **96.2** | **95.9** | **96.1** |
|  | **8** | **99.5** | **99.4** | **99.4** | **99.4** | **99.4** | **99.4** | **100.0** | **—** | **98.5** | **99.3** | **96.4** | **96.5** | **96.6** | **96.6** | **97.5** | **97.9** | **96.8** | **98.5** | **98.2** | **98.2** | **98.1** | **96.7** | **96.7** | **96.6** | **96.7** | **96.0** | **95.8** | **96.0** | **95.8** | **95.7** | **95.7** | **95.8** | **95.8** | **96.1** | **96.1** | **96.2** | **96.1** | **96.1** | **96.2** | **95.9** | **96.2** |
|  | **9** | **99.5** | **99.4** | **99.4** | **99.4** | **99.4** | **99.4** | **99.9** | **99.8** | **—** | **98.4** | **96.5** | **96.5** | **96.5** | **96.8** | **97.6** | **97.8** | **96.7** | **99.7** | **98.7** | **98.8** | **98.7** | **96.6** | **96.7** | **96.5** | **96.6** | **96.0** | **95.8** | **96.0** | **95.9** | **95.8** | **95.8** | **95.8** | **95.9** | **96.1** | **96.1** | **96.3** | **96.1** | **96.1** | **96.3** | **95.9** | **96.2** |
|  | **10** | **99.5** | **99.4** | **99.4** | **99.4** | **99.4** | **99.4** | **99.9** | **99.8** | **99.8** | **—** | **96.5** | **96.5** | **96.5** | **96.7** | **97.5** | **97.8** | **96.8** | **98.4** | **98.1** | **98.2** | **98.1** | **96.7** | **96.8** | **96.7** | **96.7** | **96.0** | **95.8** | **96.0** | **95.8** | **95.7** | **95.7** | **95.8** | **95.8** | **96.1** | **96.0** | **96.2** | **96.1** | **96.1** | **96.2** | **96.0** | **96.2** |
|  | **11** | **99.3** | **99.2** | **99.2** | **99.2** | **99.2** | **99.2** | **99.3** | **99.3** | **99.3** | **99.2** | **—** | **96.3** | **96.2** | **98.2** | **96.3** | **96.3** | **96.5** | **96.5** | **96.3** | **96.4** | **96.3** | **96.4** | **96.4** | **96.3** | **96.3** | **95.7** | **95.7** | **95.8** | **95.7** | **95.6** | **95.7** | **95.7** | **95.7** | **95.6** | **95.6** | **96.0** | **95.8** | **95.8** | **96.0** | **95.8** | **95.9** |
|  | **12** | **99.3** | **99.2** | **99.2** | **99.2** | **99.2** | **99.2** | **99.4** | **99.4** | **99.3** | **99.3** | **99.2** | **—** | **97.3** | **96.5** | **96.2** | **96.4** | **97.6** | **96.4** | **96.3** | **96.4** | **96.2** | **97.5** | **97.6** | **97.5** | **97.5** | **95.9** | **95.7** | **96.0** | **95.7** | **95.7** | **95.7** | **95.7** | **95.7** | **95.9** | **95.9** | **96.1** | **96.0** | **95.9** | **96.1** | **95.8** | **96.0** |
|  | **13** | **99.4** | **99.3** | **99.3** | **99.3** | **99.3** | **99.3** | **99.5** | **99.5** | **99.4** | **99.4** | **99.2** | **99.3** | **—** | **96.5** | **96.2** | **96.4** | **97.6** | **96.4** | **96.3** | **96.3** | **96.5** | **97.4** | **97.4** | **97.3** | **97.3** | **95.8** | **95.6** | **95.8** | **95.6** | **95.5** | **95.5** | **95.6** | **95.7** | **95.9** | **95.9** | **95.9** | **95.8** | **95.7** | **96.0** | **95.7** | **95.9** |
|  | **14** | **99.5** | **99.4** | **99.4** | **99.4** | **99.4** | **99.4** | **99.5** | **99.5** | **99.5** | **99.4** | **99.7** | **99.3** | **99.4** | **—** | **96.5** | **96.5** | **96.8** | **96.7** | **96.4** | **96.5** | **96.5** | **96.6** | **96.6** | **96.5** | **96.6** | **96.0** | **95.8** | **96.0** | **95.9** | **95.8** | **95.8** | **95.9** | **95.9** | **96.1** | **96.1** | **96.4** | **96.3** | **96.3** | **96.5** | **96.2** | **96.3** |
|  | **15** | **99.6** | **99.6** | **99.6** | **99.6** | **99.6** | **99.6** | **99.8** | **99.8** | **99.8** | **99.8** | **99.4** | **99.5** | **99.6** | **99.6** | **—** | **97.4** | **96.5** | **97.6** | **97.3** | **97.3** | **97.2** | **96.4** | **96.4** | **96.3** | **96.4** | **95.7** | **95.7** | **95.8** | **95.7** | **95.7** | **95.7** | **95.7** | **95.7** | **95.9** | **95.9** | **95.9** | **95.9** | **96.0** | **96.0** | **95.8** | **96.0** |
|  | **16** | **99.6** | **99.5** | **99.5** | **99.5** | **99.5** | **99.5** | **99.7** | **99.7** | **99.6** | **99.6** | **99.3** | **99.3** | **99.4** | **99.4** | **99.8** | **—** | **96.7** | **97.8** | **97.6** | **97.7** | **97.6** | **96.5** | **96.6** | **96.5** | **96.5** | **96.1** | **95.8** | **96.1** | **95.8** | **95.8** | **95.8** | **95.9** | **95.9** | **96.1** | **96.1** | **96.2** | **96.0** | **96.0** | **96.2** | **95.8** | **96.1** |
|  | **17** | **99.6** | **99.5** | **99.5** | **99.5** | **99.5** | **99.5** | **99.7** | **99.7** | **99.6** | **99.6** | **99.4** | **99.4** | **99.6** | **99.5** | **99.8** | **99.6** | **—** | **96.7** | **96.5** | **96.5** | **96.4** | **98.7** | **98.7** | **98.6** | **98.7** | **96.1** | **95.9** | **96.1** | **95.9** | **95.8** | **95.8** | **95.9** | **95.9** | **96.1** | **96.1** | **96.3** | **96.2** | **96.1** | **96.4** | **96.1** | **96.3** |
|  | **18** | **99.5** | **99.4** | **99.4** | **99.4** | **99.4** | **99.4** | **99.9** | **99.8** | **99.9** | **99.8** | **99.3** | **99.3** | **99.4** | **99.5** | **99.8** | **99.6** | **99.6** | **—** | **98.7** | **98.8** | **98.6** | **96.6** | **96.6** | **96.5** | **96.5** | **96.0** | **95.9** | **96.0** | **95.8** | **95.8** | **95.8** | **95.8** | **95.8** | **96.1** | **96.0** | **96.2** | **96.0** | **96.1** | **96.3** | **95.9** | **96.2** |
|  | **19** | **99.4** | **99.3** | **99.3** | **99.3** | **99.3** | **99.3** | **99.7** | **99.6** | **99.7** | **99.6** | **99.2** | **99.2** | **99.3** | **99.4** | **99.7** | **99.5** | **99.5** | **99.7** | **—** | **99.5** | **99.4** | **96.4** | **96.5** | **96.3** | **96.4** | **95.8** | **95.7** | **95.8** | **95.6** | **95.6** | **95.6** | **95.6** | **95.7** | **96.0** | **95.9** | **96.0** | **95.8** | **95.8** | **96.1** | **95.6** | **96.0** |
|  | **20** | **99.4** | **99.3** | **99.3** | **99.3** | **99.3** | **99.3** | **99.7** | **99.7** | **99.7** | **99.6** | **99.3** | **99.3** | **99.4** | **99.5** | **99.7** | **99.6** | **99.6** | **99.7** | **99.8** | **—** | **99.6** | **96.4** | **96.5** | **96.4** | **96.4** | **95.8** | **95.7** | **95.8** | **95.6** | **95.5** | **95.5** | **95.6** | **95.6** | **95.9** | **95.9** | **96.0** | **95.8** | **95.8** | **96.0** | **95.7** | **95.9** |
|  | **21** | **99.4** | **99.3** | **99.3** | **99.3** | **99.3** | **99.3** | **99.7** | **99.7** | **99.7** | **99.6** | **99.3** | **99.3** | **99.4** | **99.5** | **99.7** | **99.6** | **99.6** | **99.7** | **99.8** | **99.9** | **—** | **96.3** | **96.4** | **96.3** | **96.3** | **95.7** | **95.7** | **95.8** | **95.6** | **95.5** | **95.5** | **95.6** | **95.6** | **95.9** | **95.8** | **95.9** | **95.7** | **95.7** | **95.9** | **95.5** | **95.8** |
|  | **22** | **99.7** | **99.6** | **99.6** | **99.6** | **99.6** | **99.6** | **99.8** | **99.7** | **99.7** | **99.8** | **99.4** | **99.5** | **99.6** | **99.6** | **99.9** | **99.7** | **99.8** | **99.7** | **99.6** | **99.6** | **99.6** | **—** | **99.9** | **99.8** | **99.8** | **96.0** | **95.8** | **96.0** | **95.9** | **95.8** | **95.8** | **95.9** | **95.9** | **96.0** | **96.0** | **96.3** | **96.1** | **96.0** | **96.3** | **95.9** | **96.2** |
|  | **23** | **99.6** | **99.5** | **99.5** | **99.5** | **99.5** | **99.5** | **99.7** | **99.7** | **99.6** | **99.7** | **99.4** | **99.5** | **99.6** | **99.6** | **99.8** | **99.7** | **99.8** | **99.6** | **99.5** | **99.6** | **99.6** | **99.9** | **—** | **99.8** | **99.8** | **96.0** | **95.8** | **96.0** | **95.9** | **95.9** | **95.8** | **96.0** | **96.0** | **96.1** | **96.1** | **96.3** | **96.1** | **96.1** | **96.4** | **96.0** | **96.3** |
|  | **24** | **99.6** | **99.5** | **99.5** | **99.5** | **99.5** | **99.5** | **99.7** | **99.7** | **99.6** | **99.7** | **99.4** | **99.5** | **99.6** | **99.6** | **99.8** | **99.7** | **99.8** | **99.6** | **99.5** | **99.6** | **99.6** | **99.9** | **99.9** | **—** | **99.8** | **95.9** | **95.7** | **95.9** | **95.8** | **95.7** | **95.7** | **95.8** | **95.8** | **96.0** | **96.0** | **96.2** | **96.0** | **95.9** | **96.2** | **95.8** | **96.1** |
|  | **25** | **99.6** | **99.5** | **99.5** | **99.5** | **99.5** | **99.5** | **99.7** | **99.6** | **99.6** | **99.7** | **99.3** | **99.4** | **99.5** | **99.5** | **99.8** | **99.6** | **99.7** | **99.6** | **99.5** | **99.5** | **99.5** | **99.9** | **99.8** | **99.8** | **—** | **95.9** | **95.7** | **96.0** | **95.8** | **95.8** | **95.8** | **95.9** | **95.9** | **96.0** | **96.0** | **96.2** | **96.0** | **96.0** | **96.3** | **95.9** | **96.2** |
| **C** | **26** | **99.5** | **99.4** | **99.4** | **99.4** | **99.4** | **99.4** | **99.4** | **99.3** | **99.2** | **99.2** | **99.2** | **99.3** | **99.3** | **99.4** | **99.4** | **99.4** | **99.4** | **99.2** | **99.1** | **99.2** | **99.2** | **99.4** | **99.4** | **99.4** | **99.3** | **—** | **96.1** | **95.9** | **95.6** | **95.6** | **95.6** | **95.7** | **95.7** | **96.0** | **96.0** | **96.3** | **96.1** | **96.1** | **96.3** | **96.0** | **96.2** |
|  | **27** | **99.6** | **99.5** | **99.5** | **99.5** | **99.5** | **99.5** | **99.4** | **99.4** | **99.3** | **99.3** | **99.2** | **99.2** | **99.2** | **99.4** | **99.5** | **99.4** | **99.4** | **99.3** | **99.2** | **99.2** | **99.2** | **99.5** | **99.4** | **99.4** | **99.4** | **99.4** | **—** | **95.7** | **95.4** | **95.4** | **95.4** | **95.5** | **95.4** | **96.0** | **96.0** | **96.0** | **96.0** | **95.9** | **96.1** | **95.9** | **96.0** |
| **D** | **28** | **99.9** | **99.8** | **99.8** | **99.8** | **99.8** | **99.8** | **99.7** | **99.6** | **99.6** | **99.6** | **99.4** | **99.4** | **99.5** | **99.6** | **99.8** | **99.7** | **99.7** | **99.6** | **99.5** | **99.5** | **99.5** | **99.8** | **99.7** | **99.7** | **99.7** | **99.6** | **99.7** | **—** | **97.6** | **97.5** | **97.5** | **97.6** | **97.6** | **96.2** | **96.2** | **96.4** | **96.3** | **96.3** | **96.5** | **96.2** | **96.3** |
|  | **29** | **99.4** | **99.3** | **99.3** | **99.3** | **99.3** | **99.3** | **99.3** | **99.3** | **99.2** | **99.2** | **99.2** | **99.1** | **99.2** | **99.4** | **99.4** | **99.4** | **99.4** | **99.2** | **99.1** | **99.2** | **99.2** | **99.4** | **99.4** | **99.4** | **99.3** | **99.3** | **99.2** | **99.5** | **—** | **99.7** | **99.8** | **99.7** | **99.7** | **96.0** | **96.0** | **96.3** | **96.1** | **96.1** | **96.3** | **96.0** | **96.1** |
|  | **30** | **99.4** | **99.3** | **99.3** | **99.3** | **99.3** | **99.3** | **99.3** | **99.2** | **99.2** | **99.2** | **99.1** | **99.0** | **99.1** | **99.3** | **99.4** | **99.3** | **99.3** | **99.2** | **99.1** | **99.1** | **99.1** | **99.4** | **99.3** | **99.3** | **99.3** | **99.2** | **99.2** | **99.5** | **99.9** | **—** | **99.6** | **99.7** | **99.7** | **96.0** | **96.0** | **96.2** | **96.0** | **96.1** | **96.2** | **95.9** | **96.1** |
|  | **31** | **99.4** | **99.3** | **99.3** | **99.3** | **99.3** | **99.3** | **99.3** | **99.2** | **99.2** | **99.2** | **99.2** | **99.0** | **99.1** | **99.3** | **99.4** | **99.3** | **99.3** | **99.2** | **99.1** | **99.1** | **99.1** | **99.4** | **99.3** | **99.3** | **99.3** | **99.2** | **99.2** | **99.5** | **99.9** | **99.9** | **—** | **99.6** | **99.6** | **95.9** | **95.9** | **96.2** | **96.0** | **96.0** | **96.2** | **95.9** | **96.0** |
|  | **32** | **99.5** | **99.4** | **99.4** | **99.4** | **99.4** | **99.4** | **99.4** | **99.4** | **99.3** | **99.3** | **99.3** | **99.1** | **99.2** | **99.4** | **99.5** | **99.4** | **99.4** | **99.3** | **99.2** | **99.3** | **99.3** | **99.5** | **99.5** | **99.5** | **99.4** | **99.4** | **99.3** | **99.6** | **99.9** | **99.8** | **99.8** | **—** | **99.8** | **96.0** | **96.0** | **96.2** | **96.1** | **96.1** | **96.2** | **96.0** | **96.1** |
|  | **33** | **99.5** | **99.4** | **99.4** | **99.4** | **99.4** | **99.4** | **99.4** | **99.3** | **99.3** | **99.3** | **99.2** | **99.1** | **99.2** | **99.4** | **99.5** | **99.4** | **99.4** | **99.3** | **99.2** | **99.2** | **99.2** | **99.5** | **99.4** | **99.4** | **99.4** | **99.3** | **99.3** | **99.6** | **99.9** | **99.9** | **99.9** | **99.9** | **—** | **96.0** | **96.0** | **96.2** | **96.1** | **96.1** | **96.2** | **95.9** | **96.0** |
| **E** | **34** | **99.4** | **99.3** | **99.3** | **99.3** | **99.3** | **99.3** | **99.3** | **99.3** | **99.2** | **99.2** | **99.1** | **99.1** | **99.1** | **99.3** | **99.3** | **99.3** | **99.3** | **99.2** | **99.0** | **99.1** | **99.1** | **99.3** | **99.3** | **99.3** | **99.2** | **99.3** | **99.4** | **99.5** | **99.1** | **99.0** | **99.0** | **99.2** | **99.1** | **—** | **99.9** | **96.6** | **96.5** | **96.5** | **96.6** | **96.4** | **96.5** |
|  | **35** | **99.5** | **99.4** | **99.4** | **99.4** | **99.4** | **99.4** | **99.3** | **99.3** | **99.2** | **99.2** | **99.2** | **99.2** | **99.2** | **99.4** | **99.4** | **99.4** | **99.4** | **99.2** | **99.1** | **99.2** | **99.2** | **99.4** | **99.4** | **99.4** | **99.3** | **99.4** | **99.4** | **99.6** | **99.2** | **99.1** | **99.1** | **99.3** | **99.2** | **99.9** | **—** | **96.6** | **96.5** | **96.4** | **96.6** | **96.3** | **96.5** |
| **F** | **36** | **99.6** | **99.5** | **99.5** | **99.5** | **99.5** | **99.5** | **99.5** | **99.4** | **99.3** | **99.3** | **99.3** | **99.3** | **99.3** | **99.5** | **99.5** | **99.5** | **99.5** | **99.3** | **99.2** | **99.3** | **99.3** | **99.5** | **99.5** | **99.5** | **99.4** | **99.6** | **99.6** | **99.7** | **99.3** | **99.2** | **99.2** | **99.4** | **99.3** | **99.4** | **99.5** | **—** | **98.6** | **98.5** | **99.9** | **98.5** | **99.7** |
|  | **37** | **99.6** | **99.5** | **99.5** | **99.5** | **99.5** | **99.5** | **99.4** | **99.4** | **99.4** | **99.3** | **99.3** | **99.3** | **99.3** | **99.5** | **99.5** | **99.5** | **99.5** | **99.4** | **99.3** | **99.3** | **99.3** | **99.5** | **99.5** | **99.5** | **99.4** | **99.5** | **99.5** | **99.7** | **99.3** | **99.2** | **99.2** | **99.4** | **99.3** | **99.4** | **99.5** | **99.7** | **—** | **99.4** | **98.6** | **99.7** | **98.5** |
|  | **38** | **99.4** | **99.3** | **99.3** | **99.3** | **99.3** | **99.3** | **99.2** | **99.2** | **99.2** | **99.1** | **99.1** | **99.1** | **99.2** | **99.4** | **99.3** | **99.3** | **99.3** | **99.2** | **99.1** | **99.1** | **99.1** | **99.3** | **99.3** | **99.3** | **99.2** | **99.3** | **99.3** | **99.5** | **99.1** | **99.0** | **99.0** | **99.2** | **99.1** | **99.2** | **99.3** | **99.5** | **99.6** | **—** | **98.6** | **99.3** | **98.4** |
|  | **39** | **99.6** | **99.5** | **99.5** | **99.5** | **99.5** | **99.5** | **99.5** | **99.4** | **99.3** | **99.3** | **99.3** | **99.3** | **99.3** | **99.5** | **99.5** | **99.5** | **99.5** | **99.3** | **99.2** | **99.3** | **99.3** | **99.5** | **99.5** | **99.5** | **99.4** | **99.6** | **99.6** | **99.7** | **99.3** | **99.2** | **99.2** | **99.4** | **99.3** | **99.4** | **99.5** | **100.0** | **99.7** | **99.5** | **—** | **98.5** | **99.7** |
|  | **40** | **99.5** | **99.4** | **99.4** | **99.4** | **99.4** | **99.4** | **99.3** | **99.2** | **99.2** | **99.2** | **99.2** | **99.1** | **99.2** | **99.5** | **99.4** | **99.3** | **99.3** | **99.2** | **99.1** | **99.2** | **99.2** | **99.4** | **99.3** | **99.3** | **99.3** | **99.3** | **99.4** | **99.6** | **99.1** | **99.1** | **99.1** | **99.2** | **99.2** | **99.2** | **99.3** | **99.5** | **99.6** | **99.5** | **99.5** | **—** | **98.4** |
|  | **41** | **99.5** | **99.4** | **99.4** | **99.4** | **99.4** | **99.4** | **99.4** | **99.3** | **99.2** | **99.2** | **99.2** | **99.2** | **99.2** | **99.4** | **99.4** | **99.4** | **99.4** | **99.2** | **99.1** | **99.2** | **99.2** | **99.4** | **99.4** | **99.4** | **99.3** | **99.5** | **99.5** | **99.6** | **99.2** | **99.1** | **99.1** | **99.3** | **99.2** | **99.3** | **99.4** | **99.9** | **99.6** | **99.4** | **99.9** | **99.4** | **—** |

(A) L segment

(B) M segment

| **Sequence homology (%)** | | | | | | | | | | | | | | | | | | | | | | | | | | | | | | | | | | | | | | | | | | |
| --- | --- | --- | --- | --- | --- | --- | --- | --- | --- | --- | --- | --- | --- | --- | --- | --- | --- | --- | --- | --- | --- | --- | --- | --- | --- | --- | --- | --- | --- | --- | --- | --- | --- | --- | --- | --- | --- | --- | --- | --- | --- | --- |
| **Genotype** | | **A** | | | | | | **B** | | | | | | | | | | | | | | | | | | | **C** | **D** | | | | | | | **E** | | **F** | | | | | |
|  |  | **1** | **2** | **3** | **4** | **5** | **6** | **7** | **8** | **9** | **10** | **11** | **12** | **13** | **14** | **15** | **16** | **17** | **18** | **19** | **20** | **21** | **22** | **23** | **24** | **25** | **27** | **26** | **28** | **29** | **30** | **31** | **32** | **33** | **34** | **35** | **36** | **37** | **38** | **39** | **40** | **41** |
| **A** | **1** | **—** | **98.0** | **98.0** | **97.9** | **97.9** | **97.9** | **94.0** | **94.0** | **94.3** | **94.2** | **93.6** | **93.5** | **94.1** | **93.9** | **94.0** | **94.3** | **94.0** | **94.1** | **94.4** | **94.4** | **94.4** | **94.1** | **94.2** | **94.2** | **94.2** | **96.2** | **95.5** | **95.9** | **95.7** | **95.7** | **95.6** | **95.8** | **95.7** | **96.2** | **96.0** | **95.8** | **95.3** | **95.3** | **95.8** | **95.1** | **95.9** |
|  | **2** | **99.4** | **—** | **100.0** | **99.9** | **99.9** | **99.9** | **94.0** | **93.9** | **94.2** | **94.2** | **93.6** | **93.5** | **94.4** | **93.9** | **94.0** | **94.3** | **94.1** | **94.0** | **94.3** | **94.3** | **94.3** | **94.1** | **94.2** | **94.2** | **94.2** | **96.2** | **95.3** | **95.7** | **95.7** | **95.8** | **95.7** | **95.8** | **95.7** | **96.2** | **96.0** | **95.7** | **95.3** | **95.4** | **95.7** | **95.2** | **95.7** |
|  | **3** | **99.4** | **100.0** | **—** | **99.9** | **99.9** | **99.9** | **94.0** | **93.9** | **94.2** | **94.2** | **93.6** | **93.5** | **94.4** | **93.9** | **94.0** | **94.3** | **94.1** | **94.0** | **94.3** | **94.3** | **94.3** | **94.1** | **94.2** | **94.2** | **94.2** | **96.2** | **95.3** | **95.7** | **95.7** | **95.8** | **95.7** | **95.8** | **95.7** | **96.2** | **96.0** | **95.7** | **95.3** | **95.4** | **95.7** | **95.2** | **95.7** |
|  | **4** | **99.3** | **99.9** | **99.9** | **—** | **100.0** | **100.0** | **93.9** | **93.9** | **94.2** | **94.1** | **93.6** | **93.5** | **94.3** | **93.9** | **93.9** | **94.3** | **94.0** | **94.0** | **94.2** | **94.2** | **94.3** | **94.0** | **94.1** | **94.1** | **94.1** | **96.2** | **95.2** | **95.7** | **95.6** | **95.7** | **95.6** | **95.7** | **95.7** | **96.1** | **95.9** | **95.6** | **95.3** | **95.4** | **95.6** | **95.1** | **95.7** |
|  | **5** | **99.3** | **99.9** | **99.9** | **100.0** | **—** | **100.0** | **93.9** | **93.9** | **94.2** | **94.1** | **93.6** | **93.5** | **94.3** | **93.9** | **93.9** | **94.3** | **94.0** | **94.0** | **94.2** | **94.2** | **94.3** | **94.0** | **94.1** | **94.1** | **94.1** | **96.2** | **95.2** | **95.7** | **95.6** | **95.7** | **95.6** | **95.7** | **95.7** | **96.1** | **95.9** | **95.6** | **95.3** | **95.4** | **95.6** | **95.1** | **95.7** |
|  | **6** | **99.3** | **99.9** | **99.9** | **100.0** | **100.0** | **—** | **93.9** | **93.9** | **94.2** | **94.1** | **93.6** | **93.5** | **94.3** | **93.9** | **93.9** | **94.3** | **94.0** | **94.0** | **94.2** | **94.2** | **94.3** | **94.0** | **94.1** | **94.1** | **94.1** | **96.2** | **95.2** | **95.7** | **95.6** | **95.7** | **95.6** | **95.7** | **95.7** | **96.1** | **95.9** | **95.6** | **95.3** | **95.4** | **95.6** | **95.1** | **95.7** |
| **B** | **7** | **98.5** | **98.4** | **98.4** | **98.3** | **98.3** | **98.3** | **—** | **99.8** | **98.4** | **99.1** | **95.9** | **95.8** | **96.2** | **96.1** | **97.3** | **97.8** | **96.2** | **98.2** | **98.4** | **98.3** | **98.3** | **96.4** | **96.4** | **96.3** | **96.4** | **94.4** | **93.9** | **93.9** | **93.7** | **93.7** | **93.8** | **93.9** | **93.6** | **94.1** | **93.9** | **94.0** | **93.7** | **93.6** | **94.0** | **93.6** | **94.0** |
|  | **8** | **98.5** | **98.3** | **98.3** | **98.2** | **98.2** | **98.2** | **99.8** | **—** | **98.3** | **99.0** | **95.8** | **95.7** | **96.2** | **96.1** | **97.3** | **97.8** | **96.2** | **98.1** | **98.3** | **98.2** | **98.2** | **96.3** | **96.4** | **96.2** | **96.3** | **94.3** | **93.9** | **93.8** | **93.7** | **93.6** | **93.8** | **93.8** | **93.6** | **94.0** | **93.8** | **93.9** | **93.7** | **93.6** | **93.9** | **93.5** | **94.0** |
|  | **9** | **98.3** | **98.1** | **98.1** | **98.0** | **98.0** | **98.0** | **99.3** | **99.2** | **—** | **98.5** | **96.1** | **96.1** | **96.3** | **96.3** | **97.6** | **98.0** | **96.6** | **99.6** | **99.1** | **98.9** | **99.1** | **96.7** | **96.8** | **96.6** | **96.7** | **94.7** | **94.2** | **94.1** | **93.9** | **93.8** | **93.9** | **94.0** | **93.8** | **94.5** | **94.3** | **94.4** | **94.0** | **93.9** | **94.3** | **93.9** | **94.4** |
|  | **10** | **98.4** | **98.3** | **98.3** | **98.2** | **98.2** | **98.2** | **99.3** | **99.3** | **99.1** | **—** | **96.0** | **95.7** | **96.2** | **96.3** | **97.5** | **98.0** | **96.3** | **98.3** | **98.4** | **98.4** | **98.4** | **96.4** | **96.5** | **96.3** | **96.4** | **94.6** | **94.0** | **94.0** | **93.9** | **93.9** | **94.0** | **94.0** | **93.8** | **94.3** | **94.1** | **94.2** | **93.9** | **93.8** | **94.2** | **93.9** | **94.2** |
|  | **11** | **98.7** | **98.5** | **98.5** | **98.4** | **98.4** | **98.4** | **99.4** | **99.3** | **99.2** | **99.3** | **—** | **96.0** | **96.5** | **97.8** | **95.7** | **96.1** | **96.6** | **96.0** | **96.3** | **96.2** | **96.3** | **96.7** | **96.7** | **96.6** | **96.7** | **94.0** | **93.5** | **93.3** | **93.2** | **93.2** | **93.3** | **93.4** | **93.2** | **93.9** | **93.7** | **93.8** | **93.7** | **93.8** | **93.8** | **93.6** | **93.8** |
|  | **12** | **98.2** | **98.1** | **98.1** | **98.0** | **98.0** | **98.0** | **98.8** | **98.8** | **98.8** | **98.7** | **99.1** | **—** | **96.9** | **96.1** | **95.6** | **96.0** | **97.0** | **96.0** | **96.1** | **95.9** | **96.1** | **97.3** | **97.3** | **97.3** | **97.3** | **93.9** | **93.5** | **93.2** | **93.1** | **93.1** | **93.2** | **93.3** | **93.1** | **93.7** | **93.5** | **93.7** | **93.6** | **93.6** | **93.7** | **93.5** | **93.7** |
|  | **13** | **98.5** | **98.4** | **98.4** | **98.3** | **98.3** | **98.3** | **99.1** | **99.1** | **98.9** | **99.0** | **99.3** | **99.0** | **—** | **96.4** | **96.0** | **96.3** | **97.2** | **96.1** | **96.2** | **96.1** | **96.2** | **97.5** | **97.5** | **97.5** | **97.5** | **94.4** | **93.9** | **94.0** | **93.8** | **93.8** | **93.9** | **93.9** | **93.7** | **94.3** | **94.1** | **94.2** | **93.8** | **93.8** | **94.2** | **93.7** | **94.2** |
|  | **14** | **98.4** | **98.3** | **98.3** | **98.2** | **98.2** | **98.2** | **99.2** | **99.2** | **99.0** | **99.1** | **99.6** | **98.8** | **99.0** | **—** | **95.9** | **96.2** | **96.6** | **96.2** | **96.3** | **96.2** | **96.3** | **96.7** | **96.7** | **96.6** | **96.7** | **94.2** | **93.8** | **93.5** | **93.6** | **93.6** | **93.7** | **93.7** | **93.6** | **94.2** | **94.0** | **94.0** | **94.0** | **94.0** | **94.0** | **93.9** | **94.1** |
|  | **15** | **98.4** | **98.3** | **98.3** | **98.2** | **98.2** | **98.2** | **99.3** | **99.3** | **99.2** | **99.4** | **99.3** | **98.7** | **99.0** | **99.1** | **—** | **97.4** | **96.0** | **97.3** | **97.7** | **97.6** | **97.7** | **96.2** | **96.3** | **96.2** | **96.2** | **94.1** | **93.8** | **93.6** | **93.5** | **93.4** | **93.6** | **93.6** | **93.4** | **93.9** | **93.8** | **93.8** | **93.6** | **93.4** | **93.8** | **93.5** | **93.8** |
|  | **16** | **98.4** | **98.3** | **98.3** | **98.2** | **98.2** | **98.2** | **99.2** | **99.2** | **99.0** | **99.1** | **99.2** | **98.7** | **99.0** | **98.9** | **99.1** | **—** | **96.5** | **97.8** | **98.0** | **97.8** | **98.0** | **96.7** | **96.8** | **96.6** | **96.7** | **94.8** | **94.1** | **94.0** | **93.9** | **93.9** | **94.0** | **94.0** | **93.9** | **94.5** | **94.3** | **94.4** | **94.3** | **94.2** | **94.4** | **94.1** | **94.4** |
|  | **17** | **98.5** | **98.4** | **98.4** | **98.3** | **98.3** | **98.3** | **99.1** | **99.1** | **98.9** | **99.0** | **99.3** | **99.0** | **99.3** | **99.0** | **99.0** | **99.0** | **—** | **96.5** | **96.5** | **96.4** | **96.5** | **98.6** | **98.6** | **98.5** | **98.6** | **94.7** | **93.7** | **93.7** | **93.6** | **93.6** | **93.8** | **93.8** | **93.6** | **94.2** | **94.1** | **94.4** | **94.1** | **94.0** | **94.4** | **94.0** | **94.4** |
|  | **18** | **98.4** | **98.2** | **98.2** | **98.1** | **98.1** | **98.1** | **99.3** | **99.3** | **99.4** | **99.2** | **99.3** | **98.9** | **99.0** | **99.1** | **99.3** | **99.1** | **99.0** | **—** | **99.0** | **98.9** | **99.0** | **96.6** | **96.6** | **96.5** | **96.6** | **94.5** | **94.1** | **93.9** | **93.7** | **93.7** | **93.8** | **93.9** | **93.6** | **94.4** | **94.3** | **94.3** | **94.0** | **93.9** | **94.3** | **93.9** | **94.3** |
|  | **19** | **98.6** | **98.5** | **98.5** | **98.4** | **98.4** | **98.4** | **99.5** | **99.4** | **99.3** | **99.3** | **99.4** | **98.9** | **99.2** | **99.3** | **99.4** | **99.3** | **99.2** | **99.3** | **—** | **99.6** | **99.8** | **96.6** | **96.7** | **96.6** | **96.6** | **94.7** | **94.1** | **94.1** | **93.9** | **93.8** | **93.9** | **94.0** | **93.8** | **94.4** | **94.2** | **94.3** | **94.0** | **93.9** | **94.3** | **93.9** | **94.3** |
|  | **20** | **98.5** | **98.4** | **98.4** | **98.3** | **98.3** | **98.3** | **99.4** | **99.3** | **99.2** | **99.3** | **99.3** | **98.8** | **99.1** | **99.2** | **99.3** | **99.2** | **99.1** | **99.3** | **99.8** | **—** | **99.6** | **96.6** | **96.6** | **96.5** | **96.6** | **94.8** | **94.2** | **94.1** | **93.8** | **93.8** | **93.9** | **93.9** | **93.7** | **94.4** | **94.2** | **94.3** | **93.9** | **93.9** | **94.2** | **93.8** | **94.3** |
|  | **21** | **98.6** | **98.5** | **98.5** | **98.4** | **98.4** | **98.4** | **99.5** | **99.4** | **99.3** | **99.3** | **99.4** | **98.9** | **99.2** | **99.3** | **99.4** | **99.3** | **99.2** | **99.3** | **99.9** | **99.8** | **—** | **96.6** | **96.7** | **96.6** | **96.6** | **94.8** | **94.3** | **94.2** | **93.9** | **93.9** | **94.0** | **94.0** | **93.8** | **94.4** | **94.3** | **94.4** | **94.0** | **94.0** | **94.3** | **93.9** | **94.4** |
|  | **22** | **98.5** | **98.3** | **98.3** | **98.2** | **98.2** | **98.2** | **99.1** | **99.0** | **98.8** | **98.9** | **99.2** | **99.0** | **99.3** | **99.0** | **99.0** | **99.0** | **99.3** | **98.9** | **99.1** | **99.0** | **99.1** | **—** | **99.9** | **99.8** | **99.9** | **94.6** | **93.8** | **93.8** | **93.7** | **93.6** | **93.8** | **93.8** | **93.6** | **94.3** | **94.1** | **94.3** | **94.1** | **94.0** | **94.3** | **93.9** | **94.4** |
|  | **23** | **98.7** | **98.5** | **98.5** | **98.4** | **98.4** | **98.4** | **99.3** | **99.2** | **99.0** | **99.1** | **99.3** | **99.2** | **99.4** | **99.2** | **99.2** | **99.2** | **99.4** | **99.1** | **99.3** | **99.2** | **99.3** | **99.9** | **—** | **99.8** | **99.9** | **94.7** | **93.9** | **93.9** | **93.8** | **93.7** | **93.9** | **93.9** | **93.7** | **94.4** | **94.2** | **94.4** | **94.2** | **94.0** | **94.4** | **94.0** | **94.4** |
|  | **24** | **98.6** | **98.4** | **98.4** | **98.3** | **98.3** | **98.3** | **99.2** | **99.1** | **98.9** | **99.0** | **99.3** | **99.1** | **99.3** | **99.1** | **99.1** | **99.1** | **99.3** | **99.0** | **99.2** | **99.1** | **99.2** | **99.8** | **99.8** | **—** | **99.8** | **94.7** | **93.9** | **93.9** | **93.8** | **93.8** | **93.9** | **93.9** | **93.7** | **94.4** | **94.2** | **94.4** | **94.2** | **94.1** | **94.4** | **94.0** | **94.4** |
|  | **25** | **98.7** | **98.5** | **98.5** | **98.4** | **98.4** | **98.4** | **99.3** | **99.2** | **99.0** | **99.1** | **99.3** | **99.2** | **99.4** | **99.2** | **99.2** | **99.2** | **99.4** | **99.1** | **99.3** | **99.2** | **99.3** | **99.9** | **99.9** | **99.8** | **—** | **94.6** | **93.9** | **93.8** | **93.7** | **93.7** | **93.8** | **93.9** | **93.7** | **94.4** | **94.2** | **94.4** | **94.2** | **94.0** | **94.4** | **94.0** | **94.4** |
| **C** | **27** | **98.7** | **98.6** | **98.6** | **98.5** | **98.5** | **98.5** | **98.6** | **98.5** | **98.3** | **98.4** | **98.7** | **98.3** | **98.6** | **98.5** | **98.5** | **98.7** | **98.6** | **98.4** | **98.6** | **98.5** | **98.6** | **98.5** | **98.7** | **98.6** | **98.7** | **—** | **95.4** | **95.4** | **95.5** | **95.5** | **95.4** | **95.7** | **95.5** | **96.1** | **95.9** | **95.5** | **95.3** | **95.3** | **95.5** | **95.1** | **95.6** |
| **D** | **26** | **98.6** | **98.7** | **98.7** | **98.6** | **98.6** | **98.6** | **98.4** | **98.3** | **98.1** | **98.2** | **98.5** | **98.1** | **98.4** | **98.3** | **98.3** | **98.3** | **98.6** | **98.2** | **98.4** | **98.3** | **98.4** | **98.3** | **98.5** | **98.4** | **98.5** | **98.6** | **—** | **95.2** | **95.3** | **95.3** | **95.3** | **95.4** | **95.2** | **95.7** | **95.5** | **95.3** | **95.2** | **95.3** | **95.3** | **95.0** | **95.3** |
|  | **28** | **98.7** | **98.5** | **98.5** | **98.4** | **98.4** | **98.4** | **98.5** | **98.4** | **98.2** | **98.3** | **98.7** | **98.3** | **98.5** | **98.6** | **98.4** | **98.4** | **98.5** | **98.3** | **98.5** | **98.4** | **98.5** | **98.4** | **98.6** | **98.5** | **98.6** | **98.6** | **98.8** | **—** | **97.3** | **97.2** | **97.3** | **97.3** | **97.2** | **96.0** | **95.8** | **95.5** | **95.1** | **95.1** | **95.5** | **95.0** | **95.6** |
|  | **29** | **98.2** | **98.0** | **98.0** | **97.9** | **97.9** | **97.9** | **97.9** | **97.8** | **97.6** | **97.7** | **98.0** | **97.6** | **97.9** | **97.8** | **97.8** | **97.9** | **98.0** | **97.7** | **97.9** | **97.8** | **97.9** | **97.8** | **98.0** | **97.9** | **98.0** | **98.0** | **98.1** | **98.6** | **—** | **99.4** | **99.8** | **99.6** | **99.4** | **96.1** | **95.9** | **95.4** | **95.0** | **95.0** | **95.3** | **94.8** | **95.4** |
|  | **30** | **98.3** | **98.0** | **98.0** | **98.0** | **98.0** | **98.0** | **97.9** | **97.9** | **97.7** | **97.8** | **98.0** | **97.7** | **97.9** | **97.9** | **97.9** | **97.9** | **98.1** | **97.8** | **98.0** | **97.9** | **98.0** | **97.9** | **98.0** | **98.0** | **98.0** | **98.0** | **98.2** | **98.7** | **99.6** | **—** | **99.4** | **99.5** | **99.3** | **96.0** | **95.7** | **95.4** | **95.0** | **95.0** | **95.4** | **94.8** | **95.4** |
|  | **31** | **98.3** | **98.0** | **98.0** | **98.0** | **98.0** | **98.0** | **97.9** | **97.9** | **97.7** | **97.8** | **98.0** | **97.7** | **97.9** | **97.9** | **97.9** | **97.9** | **98.1** | **97.8** | **98.0** | **97.9** | **98.0** | **97.9** | **98.0** | **98.0** | **98.0** | **98.0** | **98.2** | **98.7** | **99.8** | **99.7** | **—** | **99.5** | **99.4** | **96.1** | **95.8** | **95.4** | **95.0** | **95.0** | **95.3** | **94.8** | **95.3** |
|  | **32** | **98.3** | **98.0** | **98.0** | **98.0** | **98.0** | **98.0** | **97.9** | **97.9** | **97.7** | **97.8** | **98.0** | **97.7** | **97.9** | **97.9** | **97.9** | **97.9** | **98.1** | **97.8** | **98.0** | **97.9** | **98.0** | **97.9** | **98.0** | **98.0** | **98.0** | **98.0** | **98.2** | **98.7** | **99.6** | **99.7** | **99.7** | **—** | **99.5** | **96.0** | **95.8** | **95.5** | **95.1** | **95.1** | **95.4** | **94.9** | **95.4** |
|  | **33** | **98.1** | **97.9** | **97.9** | **97.8** | **97.8** | **97.8** | **97.8** | **97.7** | **97.5** | **97.6** | **97.9** | **97.5** | **97.8** | **97.7** | **97.7** | **97.7** | **97.9** | **97.6** | **97.8** | **97.7** | **97.8** | **97.7** | **97.9** | **97.8** | **97.9** | **97.9** | **98.0** | **98.5** | **99.4** | **99.5** | **99.5** | **99.5** | **—** | **95.9** | **95.7** | **95.3** | **94.9** | **95.0** | **95.3** | **94.7** | **95.3** |
| **E** | **34** | **99.0** | **98.9** | **98.9** | **98.8** | **98.8** | **98.8** | **98.7** | **98.6** | **98.4** | **98.5** | **98.8** | **98.4** | **98.7** | **98.6** | **98.6** | **98.6** | **98.7** | **98.5** | **98.7** | **98.6** | **98.7** | **98.6** | **98.8** | **98.7** | **98.8** | **98.9** | **99.1** | **99.1** | **98.4** | **98.5** | **98.5** | **98.5** | **98.3** | **—** | **99.7** | **96.5** | **96.3** | **96.3** | **96.5** | **96.1** | **96.6** |
|  | **35** | **98.8** | **98.7** | **98.7** | **98.6** | **98.6** | **98.6** | **98.5** | **98.4** | **98.2** | **98.3** | **98.6** | **98.2** | **98.5** | **98.4** | **98.4** | **98.4** | **98.5** | **98.3** | **98.5** | **98.4** | **98.5** | **98.4** | **98.6** | **98.5** | **98.6** | **98.9** | **98.9** | **98.9** | **98.2** | **98.3** | **98.3** | **98.3** | **98.1** | **99.7** | **—** | **96.3** | **96.1** | **96.1** | **96.3** | **95.9** | **96.3** |
| **F** | **36** | **98.8** | **98.9** | **98.9** | **98.8** | **98.8** | **98.8** | **98.4** | **98.3** | **98.1** | **98.2** | **98.5** | **98.1** | **98.4** | **98.3** | **98.3** | **98.3** | **98.4** | **98.4** | **98.5** | **98.4** | **98.5** | **98.3** | **98.5** | **98.4** | **98.5** | **98.6** | **98.7** | **98.7** | **98.0** | **98.1** | **98.1** | **98.1** | **98.0** | **99.3** | **99.1** | **—** | **98.6** | **98.5** | **99.8** | **98.3** | **99.9** |
|  | **37** | **98.5** | **98.6** | **98.6** | **98.5** | **98.5** | **98.5** | **98.3** | **98.2** | **98.0** | **98.1** | **98.4** | **98.0** | **98.3** | **98.2** | **98.2** | **98.4** | **98.3** | **98.3** | **98.4** | **98.3** | **98.4** | **98.2** | **98.4** | **98.3** | **98.4** | **98.7** | **98.7** | **98.7** | **98.0** | **98.1** | **98.1** | **98.1** | **98.0** | **99.2** | **99.0** | **99.5** | **—** | **99.2** | **98.6** | **99.6** | **98.6** |
|  | **38** | **98.5** | **98.6** | **98.6** | **98.5** | **98.5** | **98.5** | **98.3** | **98.2** | **98.0** | **98.1** | **98.4** | **98.0** | **98.3** | **98.2** | **98.2** | **98.2** | **98.3** | **98.3** | **98.4** | **98.3** | **98.4** | **98.2** | **98.4** | **98.3** | **98.4** | **98.5** | **98.7** | **98.7** | **98.0** | **98.1** | **98.1** | **98.1** | **98.0** | **99.2** | **99.0** | **99.5** | **99.5** | **—** | **98.5** | **98.9** | **98.6** |
|  | **39** | **98.8** | **98.9** | **98.9** | **98.8** | **98.8** | **98.8** | **98.4** | **98.3** | **98.1** | **98.2** | **98.5** | **98.1** | **98.4** | **98.3** | **98.3** | **98.3** | **98.4** | **98.4** | **98.5** | **98.4** | **98.5** | **98.3** | **98.5** | **98.4** | **98.5** | **98.6** | **98.7** | **98.7** | **98.0** | **98.1** | **98.1** | **98.1** | **98.0** | **99.3** | **99.1** | **99.9** | **99.5** | **99.5** | **—** | **98.4** | **99.9** |
|  | **40** | **98.2** | **98.3** | **98.3** | **98.2** | **98.2** | **98.2** | **98.0** | **98.0** | **97.8** | **97.9** | **98.1** | **97.8** | **98.0** | **97.9** | **97.9** | **98.1** | **98.0** | **98.0** | **98.1** | **98.0** | **98.1** | **98.0** | **98.1** | **98.0** | **98.1** | **98.4** | **98.4** | **98.4** | **97.8** | **97.9** | **97.9** | **97.9** | **97.7** | **98.9** | **98.7** | **99.3** | **99.6** | **99.3** | **99.3** | **—** | **98.4** |
|  | **41** | **98.8** | **98.9** | **98.9** | **98.8** | **98.8** | **98.8** | **98.4** | **98.3** | **98.1** | **98.2** | **98.5** | **98.1** | **98.4** | **98.3** | **98.3** | **98.3** | **98.4** | **98.4** | **98.5** | **98.4** | **98.5** | **98.3** | **98.5** | **98.4** | **98.5** | **98.6** | **98.7** | **98.7** | **98.0** | **98.1** | **98.1** | **98.1** | **98.0** | **99.3** | **99.1** | **99.9** | **99.5** | **99.5** | **99.9** | **99.3** | **—** |

(C) S segment (NP)

| **Sequence homology (%)** | | | | | | | | | | | | | | | | | | | | | | | | | | | | | | | | | | | | | | | | | | |
| --- | --- | --- | --- | --- | --- | --- | --- | --- | --- | --- | --- | --- | --- | --- | --- | --- | --- | --- | --- | --- | --- | --- | --- | --- | --- | --- | --- | --- | --- | --- | --- | --- | --- | --- | --- | --- | --- | --- | --- | --- | --- | --- |
| **Genotype** | | **A** | | | | | | **B** | | | | | | | | | | | | | | | | | | | **C** | **D** | | | | | | | **E** | | **F** | | | | | |
|  |  | **1** | **2** | **3** | **4** | **5** | **6** | **7** | **8** | **9** | **10** | **11** | **12** | **13** | **14** | **15** | **16** | **17** | **18** | **19** | **20** | **21** | **22** | **23** | **24** | **25** | **26** | **27** | **28** | **29** | **30** | **31** | **32** | **33** | **34** | **35** | **36** | **37** | **38** | **39** | **40** | **41** |
| **A** | **1** | **—** | **98.0** | **98.0** | **98.0** | **98.0** | **98.0** | **95.8** | **95.9** | **95.9** | **95.8** | **95.4** | **96.3** | **95.7** | **94.6** | **95.5** | **96.9** | **96.5** | **95.9** | **95.5** | **95.3** | **95.3** | **95.8** | **95.9** | **95.9** | **95.8** | **97.2** | **97.6** | **97.4** | **96.9** | **97.0** | **96.9** | **97.0** | **97.0** | **97.4** | **97.4** | **95.8** | **95.9** | **95.8** | **95.8** | **95.7** | **95.8** |
|  | **2** | **100.0** | **—** | **100.0** | **100.0** | **100.0** | **100.0** | **95.1** | **95.3** | **95.3** | **94.9** | **95.3** | **95.7** | **95.4** | **94.4** | **95.1** | **96.2** | **95.5** | **95.3** | **94.9** | **94.6** | **94.6** | **94.9** | **95.0** | **95.0** | **94.9** | **97.2** | **97.2** | **96.5** | **96.2** | **96.3** | **96.2** | **96.3** | **96.3** | **97.2** | **97.2** | **95.8** | **95.8** | **95.7** | **95.8** | **95.5** | **95.8** |
|  | **3** | **100.0** | **100.0** | **—** | **100.0** | **100.0** | **100.0** | **95.1** | **95.3** | **95.3** | **94.9** | **95.3** | **95.7** | **95.4** | **94.4** | **95.1** | **96.2** | **95.5** | **95.3** | **94.9** | **94.6** | **94.6** | **94.9** | **95.0** | **95.0** | **94.9** | **97.2** | **97.2** | **96.5** | **96.2** | **96.3** | **96.2** | **96.3** | **96.3** | **97.2** | **97.2** | **95.8** | **95.8** | **95.7** | **95.8** | **95.5** | **95.8** |
|  | **4** | **100.0** | **100.0** | **100.0** | **—** | **100.0** | **100.0** | **95.1** | **95.3** | **95.3** | **94.9** | **95.3** | **95.7** | **95.4** | **94.4** | **95.1** | **96.2** | **95.5** | **95.3** | **94.9** | **94.6** | **94.6** | **94.9** | **95.0** | **95.0** | **94.9** | **97.2** | **97.2** | **96.5** | **96.2** | **96.3** | **96.2** | **96.3** | **96.3** | **97.2** | **97.2** | **95.8** | **95.8** | **95.7** | **95.8** | **95.5** | **95.8** |
|  | **5** | **100.0** | **100.0** | **100.0** | **100.0** | **—** | **100.0** | **95.1** | **95.3** | **95.3** | **94.9** | **95.3** | **95.7** | **95.4** | **94.4** | **95.1** | **96.2** | **95.5** | **95.3** | **94.9** | **94.6** | **94.6** | **94.9** | **95.0** | **95.0** | **94.9** | **97.2** | **97.2** | **96.5** | **96.2** | **96.3** | **96.2** | **96.3** | **96.3** | **97.2** | **97.2** | **95.8** | **95.8** | **95.7** | **95.8** | **95.5** | **95.8** |
|  | **6** | **100.0** | **100.0** | **100.0** | **100.0** | **100.0** | **—** | **95.1** | **95.3** | **95.3** | **94.9** | **95.3** | **95.7** | **95.4** | **94.4** | **95.1** | **96.2** | **95.5** | **95.3** | **94.9** | **94.6** | **94.6** | **94.9** | **95.0** | **95.0** | **94.9** | **97.2** | **97.2** | **96.5** | **96.2** | **96.3** | **96.2** | **96.3** | **96.3** | **97.2** | **97.2** | **95.8** | **95.8** | **95.7** | **95.8** | **95.5** | **95.8** |
| **B** | **7** | **99.6** | **99.6** | **99.6** | **99.6** | **99.6** | **99.6** | **—** | **99.9** | **99.3** | **97.8** | **95.4** | **96.2** | **95.7** | **94.9** | **97.0** | **98.4** | **95.8** | **99.3** | **98.6** | **98.6** | **98.6** | **95.4** | **95.5** | **95.5** | **95.4** | **95.9** | **95.8** | **95.7** | **95.1** | **95.3** | **95.1** | **95.3** | **95.3** | **96.2** | **96.2** | **95.7** | **95.4** | **95.3** | **95.7** | **95.1** | **95.7** |
|  | **8** | **99.6** | **99.2** | **99.2** | **99.2** | **99.2** | **99.2** | **100.0** | **—** | **99.5** | **98.0** | **95.5** | **96.3** | **95.8** | **95.0** | **97.2** | **98.5** | **95.9** | **99.5** | **98.8** | **98.8** | **98.8** | **95.5** | **95.7** | **95.7** | **95.5** | **96.1** | **95.9** | **95.8** | **95.3** | **95.4** | **95.3** | **95.4** | **95.4** | **96.3** | **96.3** | **95.8** | **95.5** | **95.4** | **95.8** | **95.3** | **95.8** |
|  | **9** | **99.6** | **99.2** | **99.2** | **99.2** | **99.2** | **99.2** | **100.0** | **99.6** | **—** | **98.0** | **95.5** | **96.3** | **95.8** | **95.0** | **97.4** | **98.8** | **95.9** | **100.0** | **99.1** | **99.3** | **99.3** | **95.5** | **95.7** | **95.7** | **95.5** | **96.1** | **95.9** | **95.8** | **95.3** | **95.4** | **95.3** | **95.4** | **95.7** | **96.3** | **96.3** | **95.5** | **95.3** | **95.1** | **95.5** | **95.0** | **95.5** |
|  | **10** | **99.6** | **99.2** | **99.2** | **99.2** | **99.2** | **99.2** | **100.0** | **99.6** | **99.6** | **—** | **94.9** | **95.4** | **95.7** | **94.0** | **96.7** | **97.8** | **95.3** | **98.0** | **97.0** | **97.3** | **97.3** | **94.9** | **95.0** | **95.0** | **94.9** | **95.1** | **95.5** | **95.4** | **95.1** | **95.3** | **95.1** | **95.3** | **95.3** | **95.7** | **95.7** | **94.9** | **94.7** | **94.6** | **94.9** | **94.4** | **94.9** |
|  | **11** | **99.6** | **99.2** | **99.2** | **99.2** | **99.2** | **99.2** | **100.0** | **99.6** | **99.6** | **99.6** | **—** | **96.3** | **96.9** | **97.7** | **95.7** | **96.6** | **95.9** | **95.5** | **95.1** | **95.1** | **94.9** | **95.5** | **95.7** | **95.7** | **95.5** | **95.1** | **95.3** | **94.9** | **95.3** | **95.4** | **95.3** | **95.4** | **95.4** | **96.1** | **96.1** | **94.6** | **94.6** | **94.2** | **94.6** | **94.3** | **94.6** |
|  | **12** | **99.6** | **99.6** | **99.6** | **99.6** | **99.6** | **99.6** | **100.0** | **100.0** | **100.0** | **100.0** | **100.0** | **—** | **95.8** | **95.5** | **96.1** | **97.3** | **98.8** | **96.3** | **96.2** | **95.9** | **95.9** | **98.4** | **98.5** | **98.5** | **98.4** | **96.1** | **95.8** | **95.4** | **94.9** | **95.0** | **94.9** | **95.0** | **95.0** | **96.5** | **96.5** | **95.1** | **95.1** | **95.0** | **95.1** | **94.9** | **95.1** |
|  | **13** | **99.2** | **99.2** | **99.2** | **99.2** | **99.2** | **99.2** | **99.6** | **99.6** | **99.6** | **99.6** | **99.6** | **99.6** | **—** | **96.1** | **95.7** | **96.5** | **95.7** | **95.8** | **95.4** | **95.1** | **95.1** | **95.3** | **95.4** | **95.4** | **95.3** | **95.1** | **95.8** | **95.1** | **95.7** | **95.8** | **95.7** | **95.5** | **95.5** | **95.8** | **95.8** | **94.9** | **94.9** | **94.7** | **94.9** | **94.6** | **94.9** |
|  | **14** | **98.8** | **98.8** | **98.8** | **98.8** | **98.8** | **98.8** | **99.2** | **99.2** | **99.2** | **99.2** | **99.2** | **99.2** | **98.8** | **—** | **95.0** | **95.9** | **95.1** | **95.0** | **94.6** | **94.6** | **94.3** | **94.7** | **95.0** | **94.9** | **95.0** | **94.6** | **94.7** | **94.3** | **94.4** | **94.6** | **94.4** | **94.6** | **94.6** | **95.5** | **95.5** | **94.0** | **94.0** | **93.6** | **94.0** | **93.8** | **94.0** |
|  | **15** | **99.6** | **99.6** | **99.6** | **99.6** | **99.6** | **99.6** | **100.0** | **100.0** | **100.0** | **100.0** | **100.0** | **100.0** | **99.6** | **99.2** | **—** | **98.1** | **95.7** | **97.4** | **96.7** | **96.7** | **96.7** | **95.3** | **95.4** | **95.4** | **95.3** | **95.8** | **95.5** | **95.4** | **95.1** | **95.3** | **95.1** | **95.3** | **95.3** | **96.2** | **96.2** | **95.4** | **94.9** | **94.7** | **95.4** | **94.6** | **95.4** |
|  | **16** | **99.6** | **99.6** | **99.6** | **99.6** | **99.6** | **99.6** | **100.0** | **100.0** | **100.0** | **100.0** | **100.0** | **100.0** | **99.6** | **99.2** | **100.0** | **—** | **96.9** | **98.8** | **97.8** | **98.1** | **98.1** | **96.5** | **96.6** | **96.6** | **96.5** | **97.0** | **96.9** | **96.7** | **95.9** | **96.1** | **95.9** | **96.1** | **96.1** | **97.3** | **97.3** | **96.5** | **95.9** | **95.8** | **96.5** | **95.7** | **96.5** |
|  | **17** | **99.6** | **99.6** | **99.6** | **99.6** | **99.6** | **99.6** | **100.0** | **100.0** | **100.0** | **100.0** | **100.0** | **100.0** | **99.6** | **99.2** | **100.0** | **100.0** | **—** | **95.9** | **95.8** | **95.5** | **95.5** | **99.1** | **99.2** | **99.2** | **99.1** | **95.7** | **95.7** | **95.3** | **94.7** | **94.9** | **94.7** | **94.9** | **94.9** | **96.1** | **96.1** | **95.0** | **95.1** | **95.0** | **95.0** | **94.9** | **95.0** |
|  | **18** | **99.6** | **99.2** | **99.2** | **99.2** | **99.2** | **99.2** | **100.0** | **99.6** | **100.0** | **99.6** | **99.6** | **100.0** | **99.6** | **99.2** | **100.0** | **100.0** | **100.0** | **—** | **99.1** | **99.3** | **99.3** | **95.5** | **95.7** | **95.7** | **95.5** | **96.1** | **95.9** | **95.8** | **95.3** | **95.4** | **95.3** | **95.4** | **95.7** | **96.3** | **96.3** | **95.5** | **95.3** | **95.1** | **95.5** | **95.0** | **95.5** |
|  | **19** | **99.2** | **98.8** | **98.8** | **98.8** | **98.8** | **98.8** | **99.6** | **99.2** | **99.2** | **99.2** | **99.2** | **99.6** | **99.2** | **98.8** | **99.6** | **99.6** | **99.6** | **99.2** | **—** | **99.5** | **99.5** | **95.4** | **95.5** | **95.5** | **95.4** | **95.7** | **95.5** | **95.4** | **94.9** | **95.0** | **94.9** | **95.0** | **95.3** | **96.2** | **96.2** | **95.1** | **94.9** | **94.7** | **95.1** | **94.6** | **95.1** |
|  | **20** | **99.6** | **99.2** | **99.2** | **99.2** | **99.2** | **99.2** | **100.0** | **99.6** | **99.6** | **99.6** | **99.6** | **100.0** | **99.6** | **99.2** | **100.0** | **100.0** | **100.0** | **99.6** | **99.2** | **—** | **99.7** | **95.1** | **95.3** | **95.3** | **95.1** | **95.4** | **95.3** | **95.1** | **94.6** | **94.7** | **94.6** | **94.7** | **95.0** | **95.9** | **95.9** | **95.1** | **94.9** | **94.4** | **95.1** | **94.6** | **95.1** |
|  | **21** | **99.6** | **99.2** | **99.2** | **99.2** | **99.2** | **99.2** | **100.0** | **99.6** | **99.6** | **99.6** | **99.6** | **100.0** | **99.6** | **99.2** | **100.0** | **100.0** | **100.0** | **99.6** | **99.2** | **99.6** | **—** | **95.1** | **95.3** | **95.3** | **95.1** | **95.4** | **95.3** | **95.1** | **94.6** | **94.7** | **94.6** | **94.7** | **95.0** | **95.9** | **95.9** | **94.9** | **94.6** | **94.4** | **94.9** | **94.3** | **94.9** |
|  | **22** | **99.2** | **98.8** | **98.8** | **98.8** | **98.8** | **98.8** | **99.6** | **99.2** | **99.2** | **99.2** | **99.2** | **99.6** | **99.2** | **98.8** | **99.6** | **99.6** | **99.6** | **99.2** | **98.8** | **99.2** | **99.2** | **—** | **99.6** | **99.9** | **99.7** | **95.3** | **95.5** | **95.1** | **94.6** | **94.7** | **94.6** | **94.7** | **94.7** | **95.7** | **95.7** | **94.6** | **94.7** | **94.6** | **94.6** | **94.4** | **94.6** |
|  | **23** | **99.2** | **98.4** | **98.4** | **98.4** | **98.4** | **98.4** | **99.2** | **98.8** | **98.8** | **98.8** | **98.8** | **99.2** | **98.8** | **98.4** | **99.2** | **99.2** | **99.2** | **98.8** | **98.4** | **98.8** | **98.8** | **99.2** | **—** | **99.7** | **99.6** | **95.4** | **95.7** | **95.3** | **94.7** | **94.9** | **94.7** | **94.9** | **94.9** | **95.8** | **95.8** | **94.7** | **94.9** | **94.7** | **94.7** | **94.6** | **94.7** |
|  | **24** | **99.2** | **98.8** | **98.8** | **98.8** | **98.8** | **98.8** | **99.6** | **99.2** | **99.2** | **99.2** | **99.2** | **99.6** | **99.2** | **98.8** | **99.6** | **99.6** | **99.6** | **99.2** | **98.8** | **99.2** | **99.2** | **99.6** | **99.2** | **—** | **99.9** | **95.4** | **95.7** | **95.3** | **94.7** | **94.9** | **94.7** | **94.9** | **94.9** | **95.8** | **95.8** | **94.7** | **94.9** | **94.7** | **94.7** | **94.6** | **94.7** |
|  | **25** | **99.2** | **98.8** | **98.8** | **98.8** | **98.8** | **98.8** | **99.6** | **99.2** | **99.2** | **99.2** | **99.2** | **99.6** | **99.2** | **98.8** | **99.6** | **99.6** | **99.6** | **99.2** | **98.8** | **99.2** | **99.2** | **99.6** | **99.2** | **99.6** | **—** | **95.3** | **95.5** | **95.1** | **94.6** | **94.7** | **94.6** | **94.7** | **94.7** | **95.7** | **95.7** | **94.6** | **94.7** | **94.6** | **94.6** | **94.4** | **94.6** |
| **C** | **26** | **99.6** | **99.6** | **99.6** | **99.6** | **99.6** | **99.6** | **99.2** | **99.2** | **99.2** | **99.2** | **99.2** | **99.2** | **98.8** | **98.4** | **99.2** | **99.2** | **99.2** | **99.2** | **98.8** | **99.2** | **99.2** | **98.8** | **98.4** | **98.8** | **98.8** | **—** | **96.9** | **96.5** | **95.9** | **96.1** | **95.9** | **96.1** | **96.1** | **97.0** | **97.0** | **95.9** | **95.7** | **95.5** | **95.9** | **95.4** | **95.9** |
| **D** | **27** | **100.0** | **99.6** | **99.6** | **99.6** | **99.6** | **99.6** | **99.6** | **99.2** | **99.2** | **99.2** | **99.2** | **99.6** | **99.2** | **98.8** | **99.6** | **99.6** | **99.6** | **99.2** | **98.8** | **99.2** | **99.2** | **98.8** | **98.4** | **98.8** | **98.8** | **99.6** | **—** | **99.1** | **98.2** | **98.6** | **98.2** | **98.4** | **98.4** | **98.0** | **98.0** | **96.1** | **96.2** | **96.1** | **96.1** | **95.9** | **96.1** |
|  | **28** | **99.6** | **99.2** | **99.2** | **99.2** | **99.2** | **99.2** | **99.2** | **98.8** | **98.8** | **98.8** | **98.8** | **99.2** | **98.8** | **98.4** | **99.2** | **99.2** | **99.2** | **98.8** | **98.4** | **98.8** | **98.8** | **98.4** | **98.0** | **98.4** | **98.4** | **99.2** | **99.2** | **—** | **98.1** | **98.2** | **98.1** | **98.2** | **98.2** | **97.6** | **97.6** | **95.7** | **95.8** | **95.7** | **95.7** | **95.5** | **95.7** |
|  | **29** | **100.0** | **99.6** | **99.6** | **99.6** | **99.6** | **99.6** | **99.6** | **99.2** | **99.2** | **99.2** | **99.2** | **99.6** | **99.2** | **98.8** | **99.6** | **99.6** | **99.6** | **99.2** | **98.8** | **99.2** | **99.2** | **98.8** | **98.4** | **98.8** | **98.8** | **99.6** | **99.6** | **99.2** | **—** | **99.5** | **100.0** | **99.5** | **99.5** | **97.3** | **97.3** | **95.4** | **95.5** | **95.4** | **95.4** | **95.3** | **95.4** |
|  | **30** | **100.0** | **99.6** | **99.6** | **99.6** | **99.6** | **99.6** | **99.6** | **99.2** | **99.2** | **99.2** | **99.2** | **99.6** | **99.2** | **98.8** | **99.6** | **99.6** | **99.6** | **99.2** | **98.8** | **99.2** | **99.2** | **98.8** | **98.4** | **98.8** | **98.8** | **99.6** | **99.6** | **99.2** | **99.6** | **—** | **99.5** | **99.7** | **99.7** | **97.4** | **97.4** | **95.3** | **95.4** | **95.3** | **95.3** | **95.1** | **95.3** |
|  | **31** | **100.0** | **99.6** | **99.6** | **99.6** | **99.6** | **99.6** | **99.6** | **99.2** | **99.2** | **99.2** | **99.2** | **99.6** | **99.2** | **98.8** | **99.6** | **99.6** | **99.6** | **99.2** | **98.8** | **99.2** | **99.2** | **98.8** | **98.4** | **98.8** | **98.8** | **99.6** | **99.6** | **99.2** | **100.0** | **99.6** | **—** | **99.5** | **99.5** | **97.3** | **97.3** | **95.4** | **95.5** | **95.4** | **95.4** | **95.3** | **95.4** |
|  | **32** | **99.6** | **99.2** | **99.2** | **99.2** | **99.2** | **99.2** | **99.2** | **98.8** | **98.8** | **98.8** | **98.8** | **99.2** | **98.8** | **98.4** | **99.2** | **99.2** | **99.2** | **98.8** | **98.4** | **98.8** | **98.8** | **98.4** | **98.0** | **98.4** | **98.4** | **99.2** | **99.2** | **98.8** | **99.2** | **99.2** | **99.2** | **—** | **99.7** | **97.4** | **97.4** | **95.3** | **95.4** | **95.3** | **95.3** | **95.1** | **95.3** |
|  | **33** | **100.0** | **99.6** | **99.6** | **99.6** | **99.6** | **99.6** | **99.6** | **99.2** | **99.2** | **99.2** | **99.2** | **99.6** | **99.2** | **98.8** | **99.6** | **99.6** | **99.6** | **99.2** | **98.8** | **99.2** | **99.2** | **98.8** | **98.4** | **98.8** | **98.8** | **99.6** | **99.6** | **99.2** | **99.6** | **99.6** | **99.6** | **99.2** | **—** | **97.4** | **97.4** | **95.3** | **95.4** | **95.3** | **95.3** | **95.1** | **95.3** |
| **E** | **34** | **100.0** | **99.6** | **99.6** | **99.6** | **99.6** | **99.6** | **99.6** | **99.2** | **99.2** | **99.2** | **99.2** | **99.6** | **99.2** | **98.8** | **99.6** | **99.6** | **99.6** | **99.2** | **98.8** | **99.2** | **99.2** | **98.8** | **98.4** | **98.8** | **98.8** | **99.6** | **99.6** | **99.2** | **99.6** | **99.6** | **99.6** | **99.2** | **99.6** | **—** | **100.0** | **97.2** | **97.6** | **97.4** | **97.2** | **97.3** | **97.2** |
|  | **35** | **100.0** | **99.6** | **99.6** | **99.6** | **99.6** | **99.6** | **99.6** | **99.2** | **99.2** | **99.2** | **99.2** | **99.6** | **99.2** | **98.8** | **99.6** | **99.6** | **99.6** | **99.2** | **98.8** | **99.2** | **99.2** | **98.8** | **98.4** | **98.8** | **98.8** | **99.6** | **99.6** | **99.2** | **99.6** | **99.6** | **99.6** | **99.2** | **99.6** | **100.0** | **—** | **97.2** | **97.6** | **97.4** | **97.2** | **97.3** | **97.2** |
| **F** | **36** | **99.6** | **99.2** | **99.2** | **99.2** | **99.2** | **99.2** | **99.2** | **98.8** | **98.8** | **98.8** | **98.8** | **99.2** | **98.8** | **98.4** | **99.2** | **99.2** | **99.2** | **98.8** | **98.4** | **98.8** | **98.8** | **98.4** | **98.0** | **98.4** | **98.4** | **99.2** | **99.2** | **98.8** | **99.2** | **99.2** | **99.2** | **98.8** | **99.2** | **99.2** | **99.2** | **—** | **98.8** | **98.4** | **100.0** | **98.5** | **100.0** |
|  | **37** | **100.0** | **99.6** | **99.6** | **99.6** | **99.6** | **99.6** | **99.6** | **99.2** | **99.2** | **99.2** | **99.2** | **99.6** | **99.2** | **98.8** | **99.6** | **99.6** | **99.6** | **99.2** | **98.8** | **99.2** | **99.2** | **98.8** | **98.4** | **98.8** | **98.8** | **99.6** | **99.6** | **99.2** | **99.6** | **99.6** | **99.6** | **99.2** | **99.6** | **99.6** | **99.6** | **99.2** | **—** | **99.6** | **98.8** | **99.7** | **98.8** |
|  | **38** | **100.0** | **99.6** | **99.6** | **99.6** | **99.6** | **99.6** | **99.6** | **99.2** | **99.2** | **99.2** | **99.2** | **99.6** | **99.2** | **98.8** | **99.6** | **99.6** | **99.6** | **99.2** | **98.8** | **99.2** | **99.2** | **98.8** | **98.4** | **98.8** | **98.8** | **99.6** | **99.6** | **99.2** | **99.6** | **99.6** | **99.6** | **99.2** | **99.6** | **99.6** | **99.6** | **99.2** | **99.6** | **—** | **98.4** | **99.3** | **98.4** |
|  | **39** | **99.6** | **99.2** | **99.2** | **99.2** | **99.2** | **99.2** | **99.2** | **98.8** | **98.8** | **98.8** | **98.8** | **99.2** | **98.8** | **98.4** | **99.2** | **99.2** | **99.2** | **98.8** | **98.4** | **98.8** | **98.8** | **98.4** | **98.0** | **98.4** | **98.4** | **99.2** | **99.2** | **98.8** | **99.2** | **99.2** | **99.2** | **98.8** | **99.2** | **99.2** | **99.2** | **100.0** | **99.2** | **99.2** | **—** | **98.5** | **100.0** |
|  | **40** | **99.6** | **99.2** | **99.2** | **99.2** | **99.2** | **99.2** | **99.2** | **98.8** | **98.8** | **98.8** | **98.8** | **99.2** | **98.8** | **98.4** | **99.2** | **99.2** | **99.2** | **98.8** | **98.4** | **98.8** | **98.8** | **98.4** | **98.0** | **98.4** | **98.4** | **99.2** | **99.2** | **98.8** | **99.2** | **99.2** | **99.2** | **98.8** | **99.2** | **99.2** | **99.2** | **98.8** | **99.2** | **99.2** | **98.8** | **—** | **98.5** |
|  | **41** | **99.6** | **99.2** | **99.2** | **99.2** | **99.2** | **99.2** | **99.2** | **98.8** | **98.8** | **98.8** | **98.8** | **99.2** | **98.8** | **98.4** | **99.2** | **99.2** | **99.2** | **98.8** | **98.4** | **98.8** | **98.8** | **98.4** | **98.0** | **98.4** | **98.4** | **99.2** | **99.2** | **98.8** | **99.2** | **99.2** | **99.2** | **98.8** | **99.2** | **99.2** | **99.2** | **99.6** | **99.2** | **99.2** | **99.6** | **98.8** | **—** |

(D) S segment (NSs)

| **Sequence homology (%)** | | | | | | | | | | | | | | | | | | | | | | | | | | | | | | | | | | | | | | | | | | |
| --- | --- | --- | --- | --- | --- | --- | --- | --- | --- | --- | --- | --- | --- | --- | --- | --- | --- | --- | --- | --- | --- | --- | --- | --- | --- | --- | --- | --- | --- | --- | --- | --- | --- | --- | --- | --- | --- | --- | --- | --- | --- | --- |
| **Genotype** | | **A** | | | | | | **B** | | | | | | | | | | | | | | | | | | | **C** | **D** | | | | | | | **E** | | **F** | | | | | |
|  |  | **1** | **2** | **3** | **4** | **5** | **6** | **7** | **8** | **9** | **10** | **11** | **12** | **13** | **14** | **15** | **16** | **17** | **18** | **19** | **20** | **21** | **22** | **23** | **24** | **25** | **26** | **27** | **28** | **29** | **30** | **31** | **32** | **33** | **34** | **35** | **36** | **37** | **38** | **39** | **40** | **41** |
| **A** | **1** | **—** | **97.2** | **97.2** | **97.2** | **97.2** | **97.2** | **95.0** | **95.1** | **95.1** | **95.4** | **94.3** | **95.6** | **94.6** | **94.8** | **95.6** | **95.7** | **94.3** | **94.9** | **95.1** | **95.4** | **95.4** | **94.6** | **94.6** | **94.7** | **94.7** | **96.8** | **94.4** | **94.4** | **94.6** | **94.6** | **94.7** | **94.8** | **94.3** | **94.4** | **94.3** | **93.9** | **94.0** | **94.7** | **94.0** | **93.9** | **94.0** |
|  | **2** | **98.6** | **—** | **100.0** | **100.0** | **100.0** | **100.0** | **95.1** | **95.2** | **95.0** | **95.2** | **94.3** | **95.2** | **94.0** | **94.4** | **95.0** | **95.8** | **94.2** | **94.8** | **95.0** | **95.2** | **95.2** | **94.7** | **94.7** | **94.8** | **94.8** | **96.5** | **94.3** | **94.6** | **94.3** | **94.2** | **94.4** | **94.6** | **94.1** | **94.7** | **94.3** | **94.7** | **94.6** | **95.2** | **94.8** | **94.4** | **94.8** |
|  | **3** | **98.6** | **100.0** | **—** | **100.0** | **100.0** | **100.0** | **95.1** | **95.2** | **95.0** | **95.2** | **94.3** | **95.2** | **94.0** | **94.4** | **95.0** | **95.8** | **94.2** | **94.8** | **95.0** | **95.2** | **95.2** | **94.7** | **94.7** | **94.8** | **94.8** | **96.5** | **94.3** | **94.6** | **94.3** | **94.2** | **94.4** | **94.6** | **94.1** | **94.7** | **94.3** | **94.7** | **94.6** | **95.2** | **94.8** | **94.4** | **94.8** |
|  | **4** | **98.6** | **100.0** | **100.0** | **—** | **100.0** | **100.0** | **95.1** | **95.2** | **95.0** | **95.2** | **94.3** | **95.2** | **94.0** | **94.4** | **95.0** | **95.8** | **94.2** | **94.8** | **95.0** | **95.2** | **95.2** | **94.7** | **94.7** | **94.8** | **94.8** | **96.5** | **94.3** | **94.6** | **94.3** | **94.2** | **94.4** | **94.6** | **94.1** | **94.7** | **94.3** | **94.7** | **94.6** | **95.2** | **94.8** | **94.4** | **94.8** |
|  | **5** | **98.6** | **100.0** | **100.0** | **100.0** | **—** | **100.0** | **95.1** | **95.2** | **95.0** | **95.2** | **94.3** | **95.2** | **94.0** | **94.4** | **95.0** | **95.8** | **94.2** | **94.8** | **95.0** | **95.2** | **95.2** | **94.7** | **94.7** | **94.8** | **94.8** | **96.5** | **94.3** | **94.6** | **94.3** | **94.2** | **94.4** | **94.6** | **94.1** | **94.7** | **94.3** | **94.7** | **94.6** | **95.2** | **94.8** | **94.4** | **94.8** |
|  | **6** | **98.6** | **100.0** | **100.0** | **100.0** | **100.0** | **—** | **95.1** | **95.2** | **95.0** | **95.2** | **94.3** | **95.2** | **94.0** | **94.4** | **95.0** | **95.8** | **94.2** | **94.8** | **95.0** | **95.2** | **95.2** | **94.7** | **94.7** | **94.8** | **94.8** | **96.5** | **94.3** | **94.6** | **94.3** | **94.2** | **94.4** | **94.6** | **94.1** | **94.7** | **94.3** | **94.7** | **94.6** | **95.2** | **94.8** | **94.4** | **94.8** |
| **B** | **7** | **99.3** | **99.3** | **99.3** | **99.3** | **99.3** | **99.3** | **—** | **99.8** | **98.4** | **98.3** | **95.8** | **96.5** | **95.9** | **95.9** | **97.1** | **98.0** | **96.1** | **98.2** | **98.2** | **98.4** | **98.4** | **96.1** | **96.1** | **96.3** | **96.3** | **95.9** | **95.2** | **95.1** | **95.5** | **95.4** | **95.6** | **95.7** | **95.2** | **95.2** | **94.9** | **94.8** | **95.0** | **95.2** | **94.9** | **94.9** | **94.9** |
|  | **8** | **99.3** | **99.0** | **99.0** | **99.0** | **99.0** | **99.0** | **100.0** | **—** | **98.6** | **98.4** | **95.9** | **96.6** | **96.0** | **96.0** | **97.2** | **98.1** | **96.3** | **98.4** | **98.4** | **98.6** | **98.6** | **96.3** | **96.3** | **96.4** | **96.4** | **96.0** | **95.4** | **95.2** | **95.6** | **95.5** | **95.7** | **95.8** | **95.4** | **95.4** | **95.0** | **94.9** | **95.1** | **95.4** | **95.0** | **95.0** | **95.0** |
|  | **9** | **99.0** | **98.6** | **98.6** | **98.6** | **98.6** | **98.6** | **99.7** | **99.3** | **—** | **98.4** | **96.1** | **96.8** | **96.0** | **96.3** | **97.4** | **98.1** | **96.6** | **99.8** | **98.4** | **98.6** | **98.6** | **96.5** | **96.5** | **96.6** | **96.6** | **96.0** | **95.7** | **95.6** | **95.6** | **95.5** | **95.7** | **95.8** | **95.4** | **95.8** | **95.5** | **95.4** | **95.4** | **95.6** | **95.5** | **95.2** | **95.5** |
|  | **10** | **99.0** | **98.6** | **98.6** | **98.6** | **98.6** | **98.6** | **99.7** | **99.3** | **99.0** | **—** | **96.4** | **97.1** | **96.7** | **96.5** | **97.6** | **98.3** | **96.5** | **98.2** | **98.4** | **98.4** | **98.4** | **96.7** | **96.7** | **96.8** | **96.8** | **96.3** | **95.6** | **95.6** | **95.6** | **95.5** | **95.7** | **95.8** | **95.4** | **95.8** | **95.5** | **95.5** | **95.8** | **96.0** | **95.6** | **95.7** | **95.6** |
|  | **11** | **98.3** | **98.0** | **98.0** | **98.0** | **98.0** | **98.0** | **99.0** | **98.6** | **98.3** | **98.3** | **—** | **96.1** | **96.4** | **98.1** | **96.4** | **96.6** | **95.4** | **95.9** | **96.4** | **96.4** | **96.4** | **95.8** | **95.8** | **95.9** | **95.7** | **94.7** | **94.2** | **94.2** | **94.2** | **94.1** | **94.3** | **94.4** | **94.0** | **94.8** | **94.6** | **94.4** | **94.6** | **94.8** | **94.6** | **94.4** | **94.6** |
|  | **12** | **99.0** | **99.0** | **99.0** | **99.0** | **99.0** | **99.0** | **99.7** | **99.7** | **99.3** | **99.3** | **98.6** | **—** | **96.0** | **96.0** | **96.7** | **97.4** | **96.7** | **96.6** | **96.6** | **96.8** | **96.8** | **97.3** | **97.3** | **97.4** | **97.5** | **95.6** | **94.9** | **95.1** | **95.2** | **95.1** | **95.4** | **95.5** | **95.0** | **95.6** | **95.2** | **95.0** | **95.5** | **95.7** | **95.1** | **95.1** | **95.1** |
|  | **13** | **98.0** | **98.0** | **98.0** | **98.0** | **98.0** | **98.0** | **98.6** | **98.6** | **98.3** | **99.0** | **97.6** | **98.3** | **—** | **96.4** | **95.7** | **96.5** | **95.2** | **95.8** | **96.3** | **96.3** | **96.3** | **95.7** | **95.7** | **95.8** | **95.4** | **95.0** | **94.1** | **94.3** | **94.4** | **94.2** | **94.4** | **94.6** | **94.1** | **94.9** | **94.6** | **94.3** | **94.7** | **94.9** | **94.4** | **94.6** | **94.4** |
|  | **14** | **98.6** | **98.0** | **98.0** | **98.0** | **98.0** | **98.0** | **98.6** | **98.6** | **98.3** | **98.3** | **98.3** | **98.3** | **97.3** | **—** | **96.7** | **96.7** | **95.5** | **96.0** | **96.5** | **96.5** | **96.5** | **95.7** | **95.7** | **95.8** | **95.6** | **94.8** | **94.8** | **94.6** | **94.6** | **94.4** | **94.7** | **94.8** | **94.3** | **94.9** | **94.7** | **94.6** | **94.7** | **94.9** | **94.7** | **94.6** | **94.7** |
|  | **15** | **99.7** | **99.0** | **99.0** | **99.0** | **99.0** | **99.0** | **99.7** | **99.7** | **99.3** | **99.3** | **98.6** | **99.3** | **98.3** | **99.0** | **—** | **97.7** | **95.7** | **97.2** | **97.4** | **97.6** | **97.6** | **96.1** | **96.1** | **96.3** | **96.3** | **95.6** | **95.0** | **95.0** | **95.2** | **95.1** | **95.4** | **95.5** | **95.0** | **95.6** | **95.6** | **95.0** | **95.1** | **95.6** | **95.1** | **95.0** | **95.1** |
|  | **16** | **98.6** | **98.6** | **98.6** | **98.6** | **98.6** | **98.6** | **99.3** | **99.3** | **99.0** | **99.0** | **98.3** | **99.0** | **98.3** | **98.0** | **99.0** | **—** | **96.5** | **97.8** | **98.0** | **98.2** | **98.2** | **96.9** | **96.9** | **97.1** | **97.1** | **96.1** | **95.8** | **95.8** | **95.9** | **95.8** | **96.0** | **96.1** | **95.7** | **95.9** | **95.6** | **95.4** | **95.5** | **95.9** | **95.5** | **95.4** | **95.5** |
|  | **17** | **98.6** | **98.6** | **98.6** | **98.6** | **98.6** | **98.6** | **99.3** | **99.3** | **99.3** | **99.0** | **98.3** | **99.0** | **98.0** | **98.0** | **99.0** | **98.6** | **—** | **96.4** | **96.3** | **96.5** | **96.5** | **98.0** | **98.0** | **98.1** | **97.6** | **94.8** | **94.0** | **94.0** | **94.4** | **94.2** | **94.4** | **94.6** | **94.1** | **95.0** | **94.7** | **94.2** | **94.8** | **94.8** | **94.3** | **94.7** | **94.3** |
|  | **18** | **98.6** | **98.3** | **98.3** | **98.3** | **98.3** | **98.3** | **99.3** | **99.0** | **99.3** | **98.6** | **98.0** | **99.0** | **98.0** | **98.0** | **99.0** | **98.6** | **99.0** | **—** | **98.2** | **98.4** | **98.4** | **96.3** | **96.3** | **96.4** | **96.4** | **95.8** | **95.5** | **95.4** | **95.4** | **95.2** | **95.5** | **95.6** | **95.1** | **95.6** | **95.2** | **95.1** | **95.1** | **95.4** | **95.2** | **95.0** | **95.2** |
|  | **19** | **98.6** | **98.3** | **98.3** | **98.3** | **98.3** | **98.3** | **99.3** | **99.0** | **98.6** | **99.3** | **98.0** | **99.0** | **98.6** | **98.0** | **99.0** | **98.6** | **98.6** | **98.3** | **—** | **99.8** | **99.8** | **96.5** | **96.5** | **96.6** | **96.6** | **95.8** | **95.2** | **95.1** | **95.4** | **95.2** | **95.5** | **95.6** | **95.1** | **95.4** | **95.0** | **94.9** | **95.1** | **95.6** | **95.0** | **95.0** | **95.0** |
|  | **20** | **99.0** | **98.6** | **98.6** | **98.6** | **98.6** | **98.6** | **99.7** | **99.3** | **99.0** | **99.0** | **98.3** | **99.3** | **98.3** | **98.3** | **99.3** | **99.0** | **99.0** | **98.6** | **99.3** | **—** | **100.0** | **96.7** | **96.7** | **96.8** | **96.8** | **96.0** | **95.5** | **95.4** | **95.6** | **95.5** | **95.7** | **95.8** | **95.4** | **95.6** | **95.2** | **95.1** | **95.4** | **95.8** | **95.2** | **95.2** | **95.2** |
|  | **21** | **99.0** | **98.6** | **98.6** | **98.6** | **98.6** | **98.6** | **99.7** | **99.3** | **99.0** | **99.0** | **98.3** | **99.3** | **98.3** | **98.3** | **99.3** | **99.0** | **99.0** | **98.6** | **99.3** | **100.0** | **—** | **96.7** | **96.7** | **96.8** | **96.8** | **96.0** | **95.5** | **95.4** | **95.6** | **95.5** | **95.7** | **95.8** | **95.4** | **95.6** | **95.2** | **95.1** | **95.4** | **95.8** | **95.2** | **95.2** | **95.2** |
|  | **22** | **99.3** | **99.0** | **99.0** | **99.0** | **99.0** | **99.0** | **100.0** | **99.7** | **99.3** | **99.3** | **98.6** | **99.7** | **98.6** | **98.6** | **99.7** | **99.3** | **99.3** | **99.0** | **99.0** | **99.3** | **99.3** | **—** | **99.8** | **99.9** | **99.4** | **95.2** | **94.8** | **94.8** | **95.1** | **94.9** | **95.1** | **95.2** | **94.8** | **95.5** | **95.1** | **94.7** | **95.2** | **95.2** | **94.8** | **94.9** | **94.8** |
|  | **23** | **99.3** | **99.0** | **99.0** | **99.0** | **99.0** | **99.0** | **100.0** | **99.7** | **99.3** | **99.3** | **98.6** | **99.7** | **98.6** | **98.6** | **99.7** | **99.3** | **99.3** | **99.0** | **99.0** | **99.3** | **99.3** | **99.9** | **—** | **99.9** | **99.4** | **95.2** | **94.7** | **94.7** | **94.9** | **94.7** | **94.9** | **95.0** | **94.6** | **95.5** | **95.1** | **94.7** | **95.2** | **95.2** | **94.8** | **94.9** | **94.8** |
|  | **24** | **99.3** | **99.0** | **99.0** | **99.0** | **99.0** | **99.0** | **100.0** | **99.7** | **99.3** | **99.3** | **98.6** | **99.7** | **98.6** | **98.6** | **99.7** | **99.3** | **99.3** | **99.0** | **99.0** | **99.3** | **99.3** | **99.9** | **99.8** | **—** | **99.5** | **95.4** | **94.8** | **94.8** | **95.0** | **94.8** | **95.0** | **95.1** | **94.7** | **95.6** | **95.2** | **94.8** | **95.4** | **95.4** | **94.9** | **95.0** | **94.9** |
|  | **25** | **98.6** | **98.3** | **98.3** | **98.3** | **98.3** | **98.3** | **99.3** | **99.0** | **98.6** | **98.6** | **98.0** | **99.0** | **98.0** | **98.0** | **99.0** | **98.6** | **98.6** | **98.3** | **98.3** | **98.6** | **98.6** | **99.0** | **99.0** | **99.0** | **—** | **95.4** | **94.8** | **94.8** | **94.9** | **94.8** | **95.0** | **95.1** | **94.7** | **95.7** | **95.4** | **94.9** | **95.5** | **95.5** | **95.0** | **95.1** | **95.0** |
| **C** | **26** | **99.0** | **99.0** | **99.0** | **99.0** | **99.0** | **99.0** | **99.7** | **99.7** | **99.3** | **99.3** | **98.6** | **99.3** | **99.0** | **98.3** | **99.3** | **99.3** | **99.0** | **99.0** | **99.0** | **99.3** | **99.3** | **99.7** | **99.7** | **99.7** | **99.0** | **—** | **95.6** | **95.6** | **95.6** | **95.5** | **95.7** | **95.8** | **95.6** | **95.1** | **95.0** | **94.6** | **94.7** | **94.9** | **94.7** | **94.6** | **94.7** |
| **D** | **27** | **98.3** | **98.0** | **98.0** | **98.0** | **98.0** | **98.0** | **99.0** | **98.6** | **98.6** | **98.3** | **97.6** | **98.6** | **97.6** | **97.6** | **98.6** | **98.3** | **99.0** | **98.3** | **98.0** | **98.3** | **98.3** | **98.6** | **98.6** | **98.6** | **98.0** | **98.6** | **—** | **98.4** | **97.2** | **97.1** | **97.3** | **97.4** | **96.9** | **94.7** | **94.7** | **94.9** | **94.3** | **94.7** | **94.8** | **94.2** | **94.8** |
|  | **28** | **97.6** | **97.3** | **97.3** | **97.3** | **97.3** | **97.3** | **98.3** | **98.0** | **98.0** | **97.6** | **96.9** | **98.0** | **96.9** | **96.9** | **98.0** | **97.6** | **98.3** | **97.6** | **97.3** | **97.6** | **97.6** | **98.0** | **98.0** | **98.0** | **97.3** | **98.0** | **99.0** | **—** | **97.1** | **96.9** | **97.2** | **97.3** | **96.8** | **94.7** | **94.4** | **94.7** | **94.3** | **94.7** | **94.6** | **94.2** | **94.6** |
|  | **29** | **98.0** | **97.6** | **97.6** | **97.6** | **97.6** | **97.6** | **98.6** | **98.3** | **98.0** | **98.0** | **97.3** | **98.3** | **97.3** | **97.3** | **98.3** | **98.0** | **98.3** | **97.6** | **97.6** | **98.0** | **98.0** | **98.3** | **98.3** | **98.3** | **97.6** | **98.3** | **98.6** | **98.0** | **—** | **99.7** | **99.9** | **99.8** | **99.5** | **95.6** | **95.4** | **94.3** | **94.0** | **94.4** | **94.2** | **93.9** | **94.2** |
|  | **30** | **97.3** | **96.9** | **96.9** | **96.9** | **96.9** | **96.9** | **98.0** | **97.6** | **97.3** | **97.3** | **96.6** | **97.6** | **96.6** | **96.6** | **97.6** | **97.3** | **97.6** | **96.9** | **96.9** | **97.3** | **97.3** | **97.6** | **97.6** | **97.6** | **96.9** | **97.6** | **98.0** | **97.3** | **99.7** | **—** | **99.8** | **99.7** | **99.4** | **95.5** | **95.2** | **94.2** | **93.9** | **94.3** | **94.1** | **93.8** | **94.1** |
|  | **31** | **98.0** | **97.6** | **97.6** | **97.6** | **97.6** | **97.6** | **98.6** | **98.3** | **98.0** | **98.0** | **97.3** | **98.3** | **97.3** | **97.3** | **98.3** | **98.0** | **98.3** | **97.6** | **97.6** | **98.0** | **98.0** | **98.3** | **98.3** | **98.3** | **97.6** | **98.3** | **98.6** | **98.0** | **99.9** | **99.8** | **—** | **99.9** | **99.7** | **95.7** | **95.5** | **94.4** | **94.1** | **94.6** | **94.3** | **94.0** | **94.3** |
|  | **32** | **98.0** | **97.6** | **97.6** | **97.6** | **97.6** | **97.6** | **98.6** | **98.3** | **98.0** | **98.0** | **97.3** | **98.3** | **97.3** | **97.3** | **98.3** | **98.0** | **98.3** | **97.6** | **97.6** | **98.0** | **98.0** | **98.3** | **98.3** | **98.3** | **97.6** | **98.3** | **98.6** | **98.0** | **99.8** | **99.7** | **99.9** | **—** | **99.5** | **95.8** | **95.6** | **94.6** | **94.2** | **94.7** | **94.4** | **94.1** | **94.4** |
|  | **33** | **97.6** | **97.3** | **97.3** | **97.3** | **97.3** | **97.3** | **98.3** | **98.0** | **97.6** | **97.6** | **96.9** | **98.0** | **96.9** | **96.9** | **98.0** | **97.6** | **98.0** | **97.3** | **97.3** | **97.6** | **97.6** | **98.0** | **98.0** | **98.0** | **97.3** | **98.0** | **98.3** | **97.6** | **99.5** | **99.4** | **99.7** | **99.5** | **—** | **95.4** | **95.1** | **94.1** | **93.8** | **94.2** | **94.0** | **93.7** | **94.0** |
| **E** | **34** | **99.0** | **98.6** | **98.6** | **98.6** | **98.6** | **98.6** | **99.7** | **99.3** | **99.0** | **99.0** | **98.3** | **99.3** | **98.3** | **98.3** | **99.3** | **99.0** | **99.0** | **98.6** | **98.6** | **99.0** | **99.0** | **99.3** | **99.3** | **99.3** | **98.6** | **99.3** | **98.3** | **97.6** | **98.0** | **97.3** | **98.0** | **98.0** | **97.6** | **—** | **99.7** | **96.9** | **97.1** | **97.3** | **97.1** | **96.9** | **97.1** |
|  | **35** | **99.0** | **98.6** | **98.6** | **98.6** | **98.6** | **98.6** | **99.7** | **99.3** | **99.0** | **99.0** | **98.3** | **99.3** | **98.3** | **98.3** | **99.3** | **99.0** | **99.0** | **98.6** | **98.6** | **99.0** | **99.0** | **99.3** | **99.3** | **99.3** | **98.6** | **99.3** | **98.3** | **97.6** | **98.0** | **97.3** | **98.0** | **98.0** | **97.6** | **99.7** | **—** | **96.8** | **96.9** | **97.2** | **96.9** | **96.8** | **96.9** |
| **F** | **36** | **98.0** | **97.6** | **97.6** | **97.6** | **97.6** | **97.6** | **98.6** | **98.3** | **98.0** | **98.0** | **97.6** | **98.3** | **97.3** | **97.6** | **98.3** | **98.0** | **98.0** | **97.6** | **97.6** | **98.0** | **98.0** | **98.3** | **98.3** | **98.3** | **97.6** | **98.3** | **97.3** | **96.6** | **96.9** | **96.3** | **96.9** | **96.9** | **96.6** | **98.6** | **98.6** | **—** | **98.3** | **98.3** | **99.9** | **98.2** | **99.9** |
|  | **37** | **98.0** | **97.6** | **97.6** | **97.6** | **97.6** | **97.6** | **98.6** | **98.3** | **98.0** | **98.0** | **97.6** | **98.3** | **97.3** | **97.6** | **98.3** | **98.0** | **98.0** | **97.6** | **97.6** | **98.0** | **98.0** | **98.3** | **98.3** | **98.3** | **97.6** | **98.3** | **97.3** | **96.6** | **96.9** | **96.3** | **96.9** | **96.9** | **96.6** | **98.6** | **98.6** | **99.7** | **—** | **99.3** | **98.4** | **99.7** | **98.4** |
|  | **38** | **98.0** | **97.6** | **97.6** | **97.6** | **97.6** | **97.6** | **98.6** | **98.3** | **98.0** | **98.0** | **97.6** | **98.3** | **97.3** | **97.6** | **98.3** | **98.0** | **98.0** | **97.6** | **97.6** | **98.0** | **98.0** | **98.3** | **98.3** | **98.3** | **97.6** | **98.3** | **97.3** | **96.6** | **96.9** | **96.3** | **96.9** | **96.9** | **96.6** | **98.6** | **98.6** | **99.7** | **99.7** | **—** | **98.4** | **99.2** | **98.4** |
|  | **39** | **98.0** | **97.6** | **97.6** | **97.6** | **97.6** | **97.6** | **98.6** | **98.3** | **98.0** | **98.0** | **97.6** | **98.3** | **97.3** | **97.6** | **98.3** | **98.0** | **98.0** | **97.6** | **97.6** | **98.0** | **98.0** | **98.3** | **98.3** | **98.3** | **97.6** | **98.3** | **97.3** | **96.6** | **96.9** | **96.3** | **96.9** | **96.9** | **96.6** | **98.6** | **98.6** | **99.7** | **99.7** | **99.7** | **—** | **98.3** | **100.0** |
|  | **40** | **97.6** | **97.3** | **97.3** | **97.3** | **97.3** | **97.3** | **98.3** | **98.0** | **97.6** | **97.6** | **97.3** | **98.0** | **96.9** | **97.3** | **98.0** | **97.6** | **97.6** | **97.3** | **97.3** | **97.6** | **97.6** | **98.0** | **98.0** | **98.0** | **97.3** | **98.0** | **96.9** | **96.3** | **96.6** | **95.9** | **96.6** | **96.6** | **96.3** | **98.3** | **98.3** | **99.3** | **99.7** | **99.3** | **99.3** | **—** | **98.3** |
|  | **41** | **98.0** | **97.6** | **97.6** | **97.6** | **97.6** | **97.6** | **98.6** | **98.3** | **98.0** | **98.0** | **97.6** | **98.3** | **97.3** | **97.6** | **98.3** | **98.0** | **98.0** | **97.6** | **97.6** | **98.0** | **98.0** | **98.3** | **98.3** | **98.3** | **97.6** | **98.3** | **97.3** | **96.6** | **96.9** | **96.3** | **96.9** | **96.9** | **96.6** | **98.6** | **98.6** | **99.7** | **99.7** | **99.7** | **100.0** | **99.3** | **—** |
